# Metabolic sensor AMPK licenses intestinal CD103^+^ DCs to induce T_reg_ responses

**DOI:** 10.1126/sciadv.aeb4813

**Published:** 2026-09-16

**Authors:** Thiago A. Patente, Julian van Duijvenvoorde, Graham A. Heieis, Eline C. Brombacher, Leonard R. Pelgrom, Joost Lambooij, Anna Zawistowska-Deniziak, Martina Erbì, Frank Otto, Arifa Ozir-Fazalalikhan, Sara Barnhoorn, José J. Fernández, Johan van der Reijden, Alwin J. van der Ham, Lukas J. A. C. Hawinkels, Bruno A. Guigas, José A. M. Barbuto, Rick M. Maizels, Bart Everts

**Affiliations:** ^1^ Leiden University Center for Infectious Diseases, Leiden University Medical Center, Leiden, Netherlands.; ^2^ Pathogen Immunobiology Group, Institute of Experimental Zoology, Faculty of Biology, University of Warsaw, 02-96 Warsaw, Poland.; ^3^ Department of Gastroenterology and Hepatology, Leiden University Medical Center, Leiden, Netherlands.; ^4^ Laboratory of Tumor Immunology, Department of Immunology, Institute of Biomedical Sciences, University of São Paulo, São Paulo, Brazil.; ^5^ School of Infection and Immunity, University of Glasgow, 120 University Place, G12 8TA Glasgow, UK.

## Abstract

Intestinal dendritic cells (DCs) play a central role in maintaining gut tolerance through priming of peripheral regulatory T cells (pT_reg_ cells). DC tolerogenicity has been linked to catabolic metabolism, but the role of AMP-activated kinase (AMPK), a key regulator of catabolic metabolism, in regulating intestinal tolerance remains unclear. We found high AMPK activation in intestinal DCs, and loss of AMPKα1 in CD11c-expressing cells (CD11c^ΔAMPKα1^) led to reduced frequencies of intestinal RALDH^+^ CD103^+^ cDC2s. This was associated with reduced pT_reg_ cell induction and consequently increased type 2 immunity in models of intestinal helminth infection. Correspondingly, CD103^+^ cDC2s from helminth-infected CD11c^ΔAMPKα1^ mice failed to prime T_reg_ cells ex vivo. Similarly, T_reg_ cell induction by AMPK-deficient human retinoic acid (RA)–induced tolerogenic CD103^+^ DCs was compromised. Mechanistically, AMPK underpinned RA-driven tolerogenicity by promoting RALDH activity and TGF-β secretion in a FoxO3-dependent manner, independent from metabolic reprogramming. Our findings identify AMPK as a key regulator of intestinal DC-mediated tolerance and as a therapeutic target to counter intestinal inflammatory disease.

## INTRODUCTION

Conventional dendritic cells (cDCs) are professional antigen-presenting cells that regulate the priming and maintenance of CD4^+^ and CD8^+^ T cell responses and thus play an important role in the development of adaptive immune responses (*1*). DCs are also key players in maintaining immune tolerance by preventing T cell proliferation and promoting the development of regulatory T cells (T_reg_ cells) (*2*, *3*). DC populations with tolerogenic properties (tolDCs) can be found in most peripheral tissues, where they play a crucial role in orchestrating peripheral T_reg_ cell (pT_reg_ cell) responses and in maintaining tissue-specific tolerance and homeostasis. Most insights in the biology of tolDCs have been gained from studies on DCs residing in the lamina propria of the gut, where maintenance of tolerance toward foreign antigens derived from resident microbiota and food is crucial. Here, an important driver of acquisition of tolerogenic properties by intestinal cDCs is dietary vitamin A, which through its active metabolite retinoic acid (RA), induces cDCs to express retinaldehyde dehydrogenase 2 (RALDH2), allowing them to produce RA themselves (*4*, *5*). This RA in conjunction with transforming growth factor–β (TGF-β) licenses intestinal cDCs to prime pT_reg_ cells (*6*–*8*). Both conventional type 1 DCs (cDC1) and cDC2s can contribute to the maintenance of pT_reg_ cells, although cDC2s expressing CD103 have been shown to have superior capacity to do so (*9*). More recent studies additionally implicate a role for a rare and unique subset of DCs expressing RORγt in driving pT_reg_ cell development, particularly to oral antigens during early life (*10*–*12*). Their mechanism of action and relative contribution for pT_reg_ cell homeostasis during adulthood and in gut inflammation is still unclear. The biology of tolDCs has also been extensively studied in in vitro models in which DCs can be rendered tolerogenic following exposure to immunosuppressive or modulatory compounds, including RA. Analogous to their in vivo counterparts, in vitro generated human RA-DCs display increased RALDH activity, TGF-β, and elevated CD103 expression (*13*).

Immune cell activation and function, including that of DCs, are closely linked to changes in cellular metabolism (*14*–*16*). While the metabolic requirements for the acquisition of an immunogenic phenotype by cDCs are fairly well characterized (*17*–*19*), the metabolic pathways involved in the development and function of tolDCs are less well defined (*2*, *20*). There is some evidence that tolDCs rely on oxidative phosphorylation (OXPHOS) for their tolerogenic functions (*2*, *20*), and it has been shown that inhibiting anabolic metabolism in DCs promotes the development of a tolerogenic phenotype (*21*), indicating a role for catabolic/oxidative metabolism in T_reg_ cell induction by DCs. However, most of these studies interrogated the role of metabolic programs in tolDC function in in vitro model systems, and there is currently still a paucity in our understanding of the metabolic requirements for tolerance induction by DCs in vivo.

Adenosine 5′-monophosphate–activated protein kinase (AMPK) is a key sensor of cellular energy status, restoring energy homeostasis upon nutrient deprivation by inhibiting anabolic pathways and promoting catabolic pathways (*22*, *23*). There is some data to suggest a role for AMPK in shaping the tolerogenic properties of DCs. For instance, human DCs treated with vitamin D3 display increased AMPK activation, which is associated with increased mitochondrial activity (*24*) and elevated mitochondrial glucose oxidation (*25*). We recently reported that AMPK activation itself is sufficient to render human DCs tolerogenic in a mechanism dependent on RALDH and supported by metabolic reprogramming involving fatty acid oxidation (FAO) and glucose catabolism (*26*), pointing to a key role for AMPK signaling in in vitro tolDC differentiation. In vivo, AMPKα1 deficiency in CD11c-expressing cells was shown to hamper host defense against *Nippostrongylus brasiliensis* infection in mice, a feature associated with increased interleukin-12 (IL-12)/23p40 secretion and poor induction of a type 2 immune response (*27*). Concomitantly, murine bone marrow–derived DCs (BMDCs) from AMPK knockout mice display an increased inflammatory phenotype characterized by an enhanced ability to induce interferon-γ (IFN-γ)– and IL-17–secreting T cells (*28*). While these studies suggest that AMPK signaling in DCs is involved in dampening their pro-inflammatory functions, the exact role played by this kinase in tolDC biology in the gut remains an open question.

Here, we set out to characterize the metabolic properties of intestinal cDCs and to define the role of AMPK signaling in their differentiation and ability to induce T_reg_ cells. We show that murine intestinal cDCs, specifically CD103^+^ cDC2s, displayed high AMPK activation, associated with increased RALDH activity, which was abrogated upon loss of AMPK signaling. Concomitantly, in vitro human RA-induced tolDCs (RA-DCs) were characterized by increased AMPK signaling, required for the induction of functional T_reg_ cells. We found AMPK to mediate this through FoxO3-driven increased RALDH activity and TGF-β expression but independently from metabolic rewiring. Furthermore, we link the loss of AMPK in cDCs to impaired T_reg_ cell induction and, consequently, exacerbated type 2 immunity during infection with the parasitic helminths *Heligmosomoides polygyrus* and *Schistosoma mansoni.* Together, these data identify a key role for AMPK signaling in controlling regulatory responses by DCs in the gut.

## RESULTS

### AMPKα1 signaling governs maintenance and RALDH activity of intestinal CD103^+^ cDC2s

**Fig. 1.**
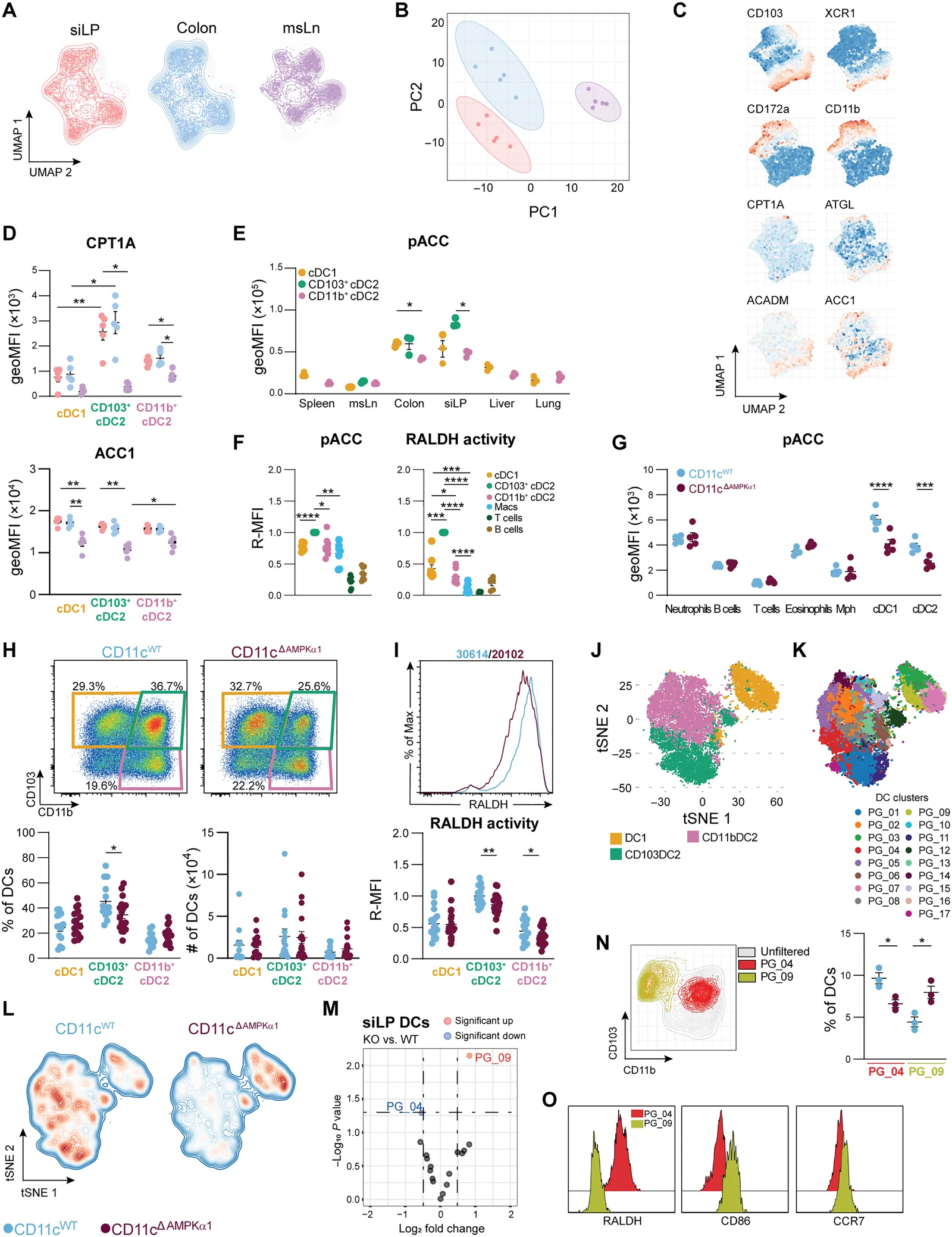
AMPKα1 signaling governs maintenance and RALDH activity of intestinal CD103^+^ cDC2s. (**A**) UMAP of intestinal DCs subsets based on expression of activation and lineage markers. (**B**) PCA of intestinal cDCs determined by the expression pattern of metabolic and activation markers. (**C**) UMAPs displaying the expression of lineage and fatty acid-related proteins. (**D**) Geometric mean fluorescence intensity (GeoMFI) of CPT1A and ACC1 in intestinal cDCs subsets. (**E**) GeoMFI of phosphorylation level of ACC at Ser^79^ (pACC) in cDCs from different tissues. (**F**) Relative geometric MFI (R-MFI) of pACC and RALDH activity, as determined by Aldefluor assay, in different immune cells in siLP of naïve WT mice, relative to CD103^+^ cDC2s. (**G**) As in (E) in spleen of naïve CD11c^WT^ and CD11c^ΔAMPKα1^ mice. (**H**) Representative dot plot, frequency, and numbers of siLP DC subsets in CD11c^WT^ and CD11c^ΔAMPKα1^ mice. (**I**) Representative histogram (up) and R-MFI of RALDH activity in siLP cDC subsets (bottom). (**J**) Opt-SNE analysis of siLP cDC subsets. (**K**) Phenograph clustering performed on RALDH, activation and lineage markers from siLP cDCs. (**L**) Contour plots overlaid on opt-SNE analysis as shown in (J) displaying distribution of cells for CD11c^WT^ and CD11c^ΔAMPKα1^ mice. (**M**) Volcano plot with significant differences in cluster frequency from (K) between CD11c^WT^ and CD11c^ΔAMPKα1^ mice. (**N**) Contour plot of PG_04 and PG_09 displaying CD103 and CD11b expression. (**O**) Histograms of indicated markers in PG_04 and PG_09. Data are representative of one of the two [(A) to (D) and (J) to (O)] and three [(E) to (G)] independent experiments or represent a pool of five to seven independent experiments [(H) and (I)]. Two-way analysis of variance (ANOVA) for repeated measures with Sidak posttest (D) or one-way ANOVA with Tukey’s HSD posttest [(E) and (F)] or Student’s *t* test [(G) to (I)] was used to assess statistically significant differences. Means ± SEM are indicated in the graphs. *P < 0.05, **P < 0.01, ***P < 0.001, and ****P < 0.0001.

To start characterizing the metabolic profiles of intestinal cDCs we analyzed cDCs isolated from colon, small intestine lamina propria (siLP) and mesenteric lymph nodes (msLn) (fig. S1) by flow cytometry to simultaneously evaluate the expression of lineage and activation markers and of metabolic enzymes from core metabolic pathways (*29*, *30*). Unbiased and principal components analysis (PCA) encompassing activation and lineage cDC markers revealed a similar profile between gut tissue resident cDCs that was distinct from migratory cDCs (migDCs) present in msLn (Fig. 1, A and B). The analysis of metabolic enzyme expression revealed that particularly the expression of fatty acid metabolism-related proteins (ACC1, ACADM, ATGL, and CPT1A) was elevated in gut tissue–resident cDCs compared to migDCs (Fig. 1, C and D, and fig. S2A), of which carnitine palmitoyl transferase 1A (CPT1A), a rate-limiting FA transporter for FAO, showed the highest expression in CD103^+^ cDC2s (Fig. 1D and fig. S2A). This would be in line with an increased catabolic metabolic profile in gut tissue–resident CD103^+^ cDC2s.

Because AMPK is one of the key proteins regulating catabolic metabolism, we next aimed to test whether AMPK signaling aligned with these findings. To assess this, we measured the phosphorylation levels of ACC1, a key downstream target of AMPK (*22*). Largely consistent with the CPT1A expression data, increased AMPK signaling was observed in cDCs from colon and siLP and particularly in siLP CD103^+^ cDC2s of naïve mice compared to cDCs from other tissues (Fig. 1E and fig. S2B) or other immune cell subsets from the gut (Fig. 1F). In these cDCs, AMPK signaling levels were positively correlated with RALDH activity (Fig. 1F), an important effector enzyme involved in tolerance induction by cDCs (*4*–*7*), and previously associated with increased AMPK signaling in DCs (*26*). To better understand how AMPK signaling affects intestinal cDC homeostasis in general, and in CD103^+^ cDC2s in particular, we generated mice with a specific deletion of AMPKα1—the primary isoform expressed by immune cells (*31*)—in CD11c+ cells (CD11c^ΔAMPKα1^), which show a defect in AMPK expression and signaling in cDCs, but not other immune cell types (Fig. 1G and fig. S2C). Mice lacking AMPK signaling in CD11c^+^ cells displayed a select number of metabolic alterations in the cDC compartment from colon, siLP, and msLn, which was largely restricted to fatty acid metabolism. Specifically, there were reduced CPT1A levels in cDC1s from the msLn, lower ACC1 levels in colonic cDC1s, and decreased ATGL, ACC1, and CD36 in colonic CD11b^+^ cDC2s (fig. S2, D and E). These alterations in FA metabolism in DCs from CD11c^ΔAMPKα1^ mice did not significantly affect mitochondrial or glycolytic dependence on adenosine 5′-triphosphate (ATP) synthesis across DC subsets as determined by SCENITH (fig. S2F) (*32*). Although siLP CD103^+^ cDC2s from CD11c^ΔAMPKα1^ mice were not strongly metabolically altered, a selective reduction in their cell frequency, but not cell numbers, could be observed (Fig. 1H). In addition, lower RALDH activity in cDC2 subsets (Fig. 1I) was observed in the siLP of CD11c^ΔAMPKα1^ mice, suggesting a role for AMPK in their homeostasis and function. Reduced frequencies of CD103^+^ cDC2s in CD11c^ΔAMPKα1^ mice were mirrored in the draining msLn (fig. S2G), although RALDH activity was not affected in these cDCs by loss of AMPKα1 (fig. S2H). To further characterize the consequences of loss of AMPKα1 on siLP cDC populations in a more unbiased manner, we performed a dimensional reduction and unsupervised analysis based on a flow cytometry panel encompassing activation and lineage markers of the intestinal cDC compartment. This resulted in the identification of several phenotypically distinct intestinal cDC clusters (Fig. 1, J and K, and fig. S2I). Among those, cluster PG_04, a CD103^+^ cDC2 population characterized by high RALDH activity and low CD86 and CCR7 expression was significantly reduced in frequency in CD11c^ΔAMPKα1^ mice, confirming the findings based on manual gating. Conversely, a RALDH^lo^CD86^hi^CCR7^hi^ cDC1 population (PG_09) was increased in frequency (Fig. 1, L to O, and fig. S2J), suggesting that multiple intestinal cDC subsets are phenotypically affected by loss of AMPK, collectively characterized by a switch from a tolerogenic to a more immunogenic phenotype in naïve CD11c^ΔAMPKα1^ mice.

We also analyzed the lung as another mucosal site with regulatory characteristics where RALDH^+^ cDCs have been identified (*33*). We did not observe any significant changes in frequencies of lung cDCs or macrophages (Mph). However, both cDC1s and alveolar macrophages (Alv Mphs) displayed reduced RALDH activity at the population level in the lungs of CD11c^ΔAMPKα1^ compared to CD11c^WT^ mice (fig. S3, A to C), reinforcing the link between AMPK signaling and RALDH activity in cDCs, and potentially Alv Mphs. When analyzing the lung cDC compartment in an unbiased manner, we identified several phenotypic clusters within the cDC1s and cDC2s subsets with different abundance between CD11c^WT^ and CD11c^ΔAMPKα1^ mice (fig. S3, D to F). Similar to our findings in the gut, we observed a shift from a tolerogenic to an immunogenic DC phenotype in CD11c^ΔAMPKα1^ mice, as illustrated by, on the one hand, a reduced frequency of a cDC1 cluster, with high RALDH activity and low expression of CD80 and CD86 (PG_04), and, on the other hand, an accumulation of a RALDH^lo^CD86^hi^CD80^hi^ cDC1 population (PG_07) (fig. S3, G to J), highlighting the importance of AMPK signaling in keeping cDCs in a quiescent state at mucosal sites. Together, these data suggest that AMPKα1 signaling controls mucosal cDCs homeostasis and governs RALDH activity.

### Human tolerogenic RA-DCs display increased AMPK signaling

**Fig. 2.**
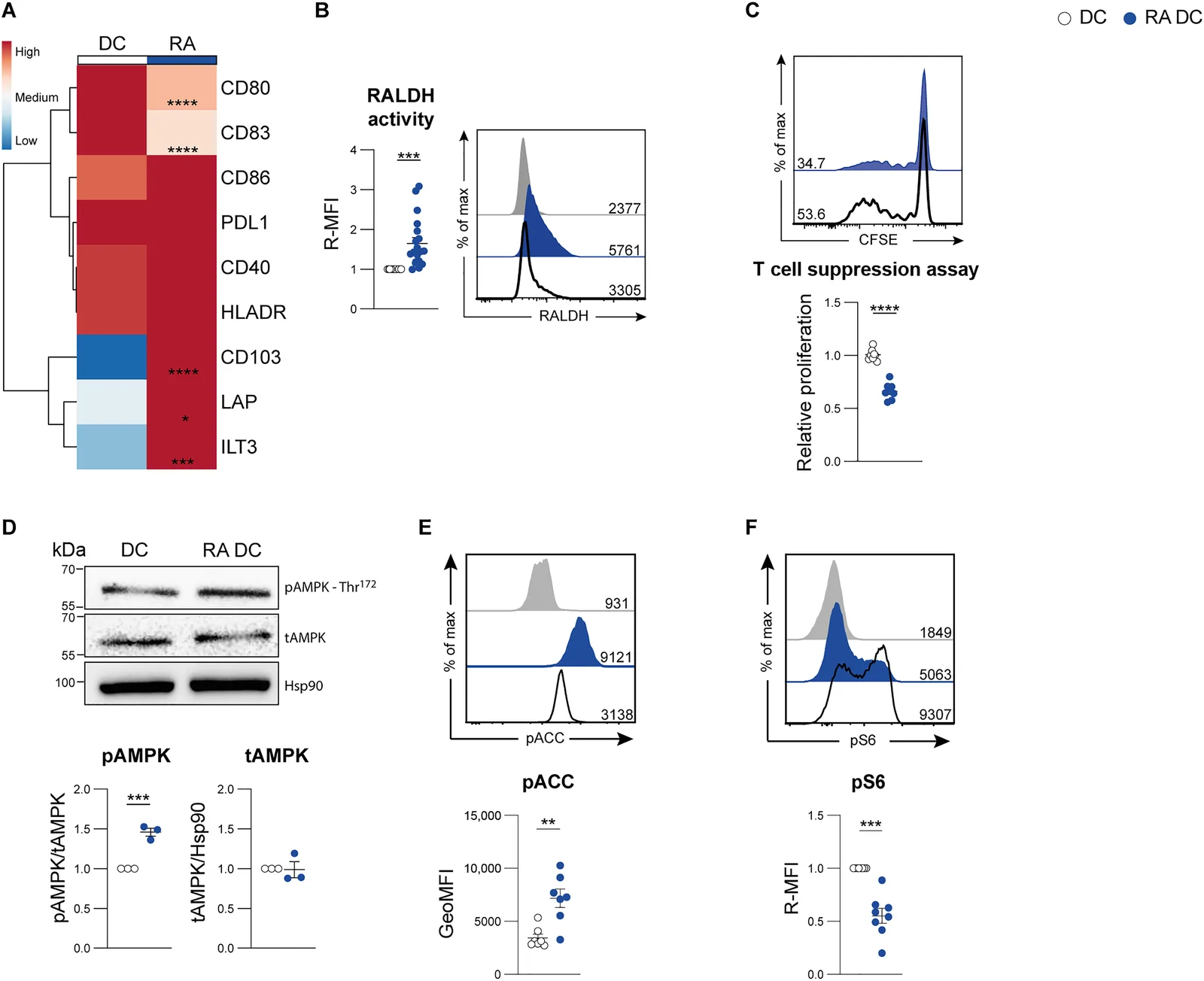
RA renders human DCs tolerogenic and induces AMPK activation. Human monocytes were isolated from PBMCs with CD14^+^ magnetic beads and differentiated into moDCs in the presence of GM-CSF + IL-4. On day 5, cells were treated with RA. On the sixth day, LPS (100 ng/ml) was added, and 24 hours later, cells were harvested for functional assays. (**A**) Heatmap of the log_2_ fold change in GeoMFI of surface markers of moDCs measured by flow cytometry relative to control-stimulated DCs (DC). (**B**) Relative GeoMFI (R-MFI) of RALDH activity in moDCs measured by flow cytometry. (**C**) T cell suppression assay was performed by coculturing irradiated Th cells derived from the DC-T cell coculture with carboxyfluorescein diacetate succinimidyl ester (CFSE)–labeled memory T_H_ cells from the same donor. On the sixth day, the CFSE dilution was measured by flow cytometry. (**D**) Representative immunoblot of the expression of phosphorylated AMPK, total AMPK, and Hsp90 in DCs either left alone or treated with RA for 48 hours. (**E** and **F**) Intracellular levels of phosphorylated ACC or S6 protein in DCs either left alone or treated with RA for 48 hours or 30 min, respectively. Data are pooled from 15 to 22 donors [(A) and (B)], 8 donors [(C), (E), and (F)], 3 donors (D), and 7 donors (E). Means ± SEM are indicated in the graphs. Paired *t* test was used to assess statistically significant differences. *P < 0.05, **P < 0.01, ***P < 0.001, and ****P < 0.0001.

To better understand if and how AMPK could be controlling the tolerogenic phenotype of intestinal DCs, we turned to an in vitro model using human moDCs exposed to RA (RA-DCs) to promote the differentiation of tolDCs resembling intestinal tolDCs characterized by high CD103 expression and RALDH activity (*13*, *34*). RA significantly increased the expression of ILT3, a co-inhibitory molecule involved in controlling the inflammatory response (*35*), the expression of the integrin CD103 (*13*), and the expression of latency-associated peptide (LAP), a proxy for TGF-β secretion (Fig. 2A and fig. S4A), as well as RALDH activity (Fig. 2B). CD4^+^ T cells cocultured with RA-DCs increased the expression of regulatory T cell markers FoxP3 (fig. S4, B and C), CTLA-4 (fig. S4D), and IL-10 (fig. S4E). Correspondingly, these T cells showed increased capacity to suppress bystander T cell proliferation (Fig. 2C), demonstrating RA-DCs induce functional T_reg_ cells.

Following the validation of their tolerogenic phenotype and function, we next investigated the metabolic profile of RA-DCs. Mirroring the siLP CD103^+^ cDC2s, RA-treated human cDCs displayed enhanced AMPK activation, as determined by its phosphorylation at Thr^172^ (Fig. 2D) and the phosphorylation of its downstream target ACC (Fig. 2E). This was accompanied by reduced phosphorylation of S6, one of the direct downstream targets of mammalian target of rapamycin complex 1 (mTORC1), and which is inhibited by AMPK signaling (Fig. 2F) (*22*, *36*). Together, these data show that RA directly promotes AMPK activation.

### RA-DCs depend on AMPK signaling for induction of T_reg_ cells

**Fig. 3.**
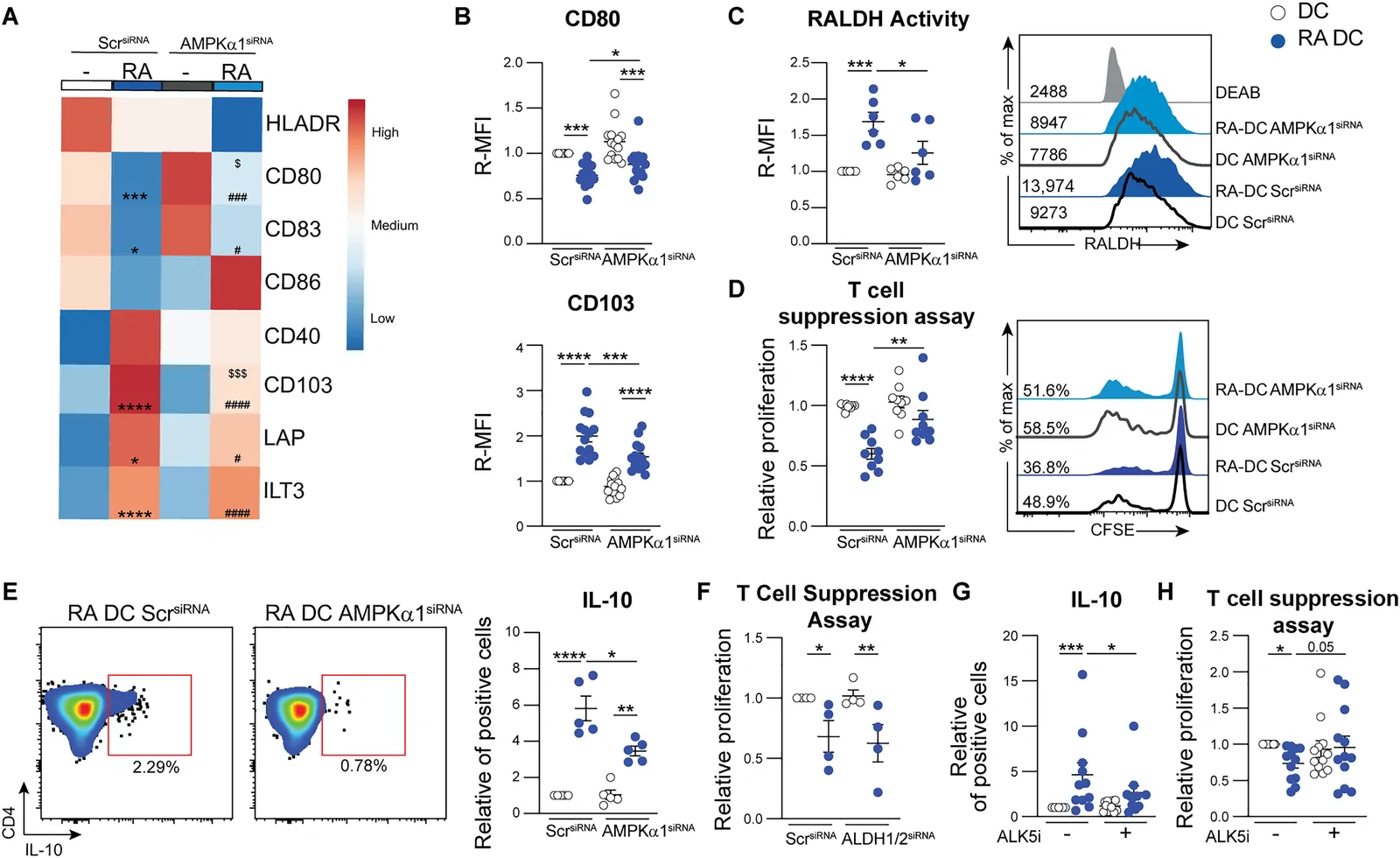
Human RA-DCs depend on AMPK signaling for induction of regulatory T cells. Human moDCs were silenced for AMPKα1 or nontargeting scramble siRNA before treatment with RA (A to E). (**A**) Heatmap of log_2_ fold change in GeoMFI of surface markers of moDCs relative to scramble control-stimulated DCs. (**B**) Relative GeoMFI (R-MFI) for the expression of indicated markers measured by flow cytometry. (**C**) Representative histogram of staining for RALDH activity and quantification of R-MFI. (**D**) T cell suppression assay as described in Fig. 2. (**E**) Intracellular levels of IL-10 measured by flow cytometry of allogeneic naïve T cells cocultured with indicated moDCs. (**F**) T cell suppression assay as described in Fig. 2. (**G** and **H**) moDCs were treated with activin A receptor type II-like kinase (ALK5) inhibitor prior to RA treatment and cocultured with naïve allogeneic T cells and the frequency of (G) IL-10^+^ T cells, and the (H) suppressive capacity of primed T_reg_ cells were evaluated. Data are pooled from 4 to 14 donors Means ± SEM are indicated in the graphs. Two-Way ANOVA for repeated measurements with Sidák’s posttest was used to assess statistically significant differences. *P < 0.05, **P < 0.01, ***P < 0.001, and ****P < 0.0001. (A) * represents significance versus scramble control-stimulated DCs (−). # represents significance versus control-stimulated-DCs AMPKα1^siRNA^. $ represents significance versus scramble RA-DCs.

To evaluate the role of AMPK signaling in phenotype and function of human RA-DCs, we silenced AMPKα1 before treatment with RA (fig. S4F). Mirroring the murine in vivo data, we found AMPKα1 signaling to be important for the increase in both CD103 expression and RALDH activity induced by RA (Fig. 3, A to C). In addition, a small but consistent reduction in LAP surface and TGF-β release was observed in RA-DCs lacking AMPK signaling, suggesting also TGF-β expression to be, in part, dependent on AMPK signaling in human RA-DCs (fig. S4, G and H). Next, we investigated the effects of AMPKα1 signaling on the ability of RA-DCs to induce the differentiation of functional T_reg_ cells and found that AMPKα1 silencing significantly compromised the ability of RA-DCs to do so (Fig. 3D). This was associated with reduced IL-10 production by these T cells (Fig. 3E). Both RALDH activity and TGF-β have been implicated in promoting T_reg_ cells by RA-DCs (*6*, *37*). The silencing of ALDH1A1 and ALDH1A2 subunits in human RA-DCs abrogated RA-induced RALDH activity (fig. S4I), but did not affect the ability of these DCs to induce functional T_reg_ cells (Fig. 3F). Instead, we found the inhibition of TGF-β signaling in human RA-DC/T cell cocultures to revert both the increased frequency of IL-10-producing T cells (Fig. 3G) and the ability of these T cells to suppress the proliferation of bystander T cells (Fig. 3H).

**Fig. 4.**
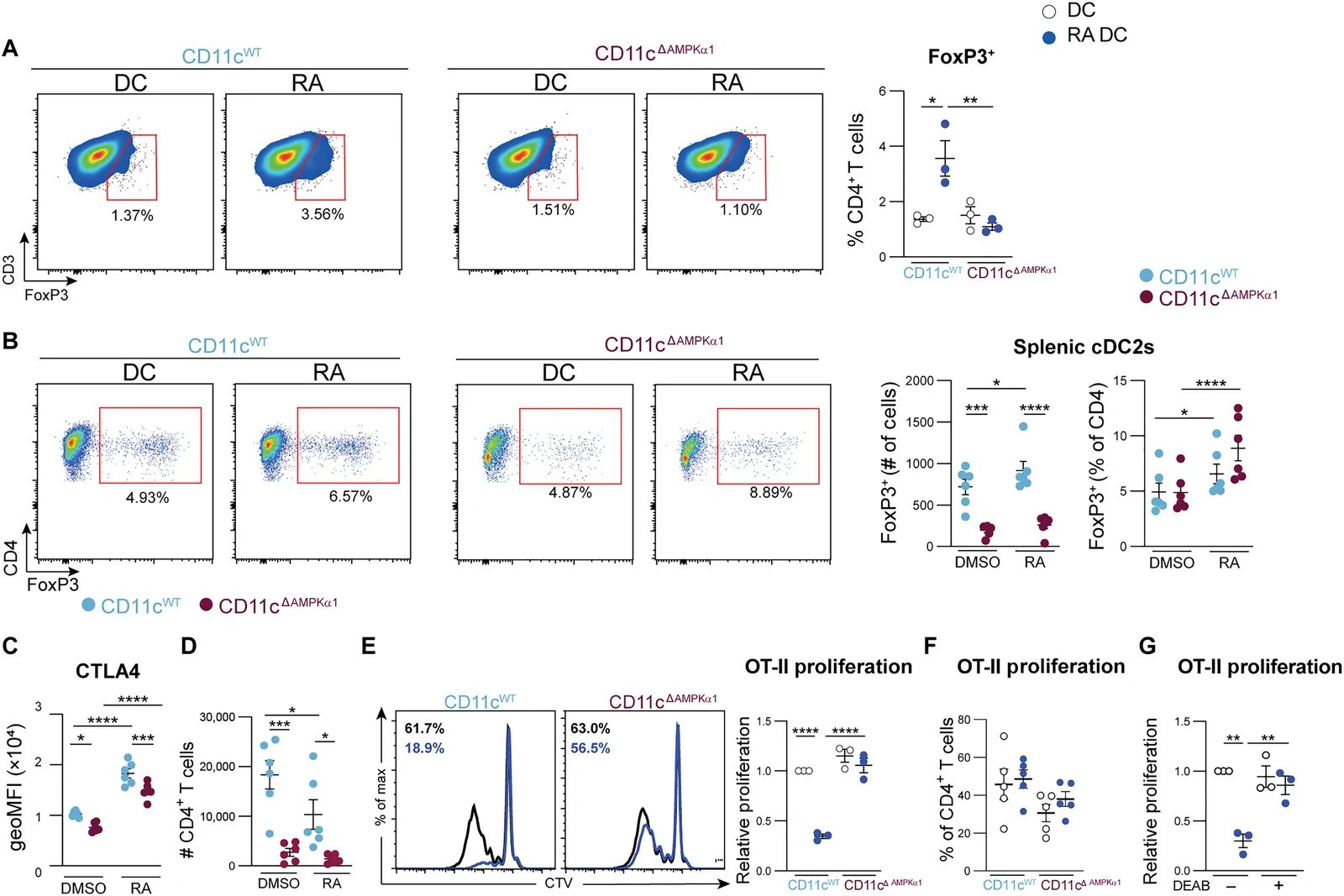
Murine RA-DCs depend on AMPK signaling for induction of regulatory T cells. (**A**) GM-CSF–derived DCs (BMDCs) from CD11c^WT^ or CD11c^ΔAMPKα1^ mice were treated with RA for 24 h followed by 24 h with LPS and OVA and cocultured with naïve OT-II T cells to evaluate their ability to induce FoxP3^+^ T_reg_ cells. Representative flow cytometry plots of percentage of FoxP3^+^ T_reg_ cells of CD4^+^ T cells. (**B** to **D**) Splenic cDC2s were sorted from the spleen of CD11c^WT^ or CD11c^ΔAMPKα1^ mice by flow cytometry treated with RA for 2 hours (in the presence of LPS, CpG-B, and OVA) and cocultured with naïve OT-II T cells. (B) Representative flow cytometry plots of percentage of FoxP3^+^ T_reg_ cells of CD4^+^ T cells and total number T_reg_ cells in cultures are shown. (C) GeoMFI of CTLA4 expression in FoxP3^+^ T_reg_ cells primed by splenic cDC2s. (D) Total number of CD4^+^ T cells after coculture with OVA-pulsed splenic cDC2s. (**E**) GM-CSF–derived BMDCs (GM-DCs) (**F**) and Flt3L-derived bone marrow-DCs (FLT3-DCs) from CD11c^WT^ or CD11c^ΔAMPKα1^ mice were stimulated as in (I) and cocultured with naïve OT-II T cells to evaluate their ability to induce OT-II T cell proliferation by CTV labeling. (**G**) T cell proliferation as determined in (E) was assessed in the presence or absence of RALDH inhibitor *N*,*N*-diethylaminobenzaldehyde (DEAB). Data are a pool of three independent experiments with one mouse of each genotype [(A), (E), and (G)], from one experiment with five (F) or six mice [(B) to (D)] per genotype. Means ± SEM are indicated in the graphs. Two-way ANOVA for repeated measurements with Sidák’s posttest was used to assess statistically significant differences. *P < 0.05, **P < 0.01, ***P < 0.001, and ****P < 0.0001.

To extend these findings, we generated murine RA-treated granulocyte-macrophage colony-stimulating factor (GM-CSF)–derived BMDCs from CD11c^∆AMPKα1^ mice and assessed their ability to promote differentiation of FoxP3^+^ T_reg_ cells. In line with the human DC data, AMPKα1 deficiency impaired their ability to up-regulate Foxp3 in cocultured CD4^+^ T cells (Fig. 4A). Also, the ability of RA-treated splenic cDC2s to enhance the total number of FoxP3^+^ T_reg_ cells and CTLA4 expression in T cell cocultures was lost in the absence of AMPK signaling, although FoxP3^+^ T_reg_ cell frequencies were not affected (Fig. 4, B and C). This appeared to be in part a consequence of reduced T cell priming by the AMPK-deficient RA-treated splenic cDC2s (Fig. 4D) and seemed to be specific to splenic DCs as AMPK deficient RA-treated GM-CSF–derived or Flt3L-derived BMDCs did not show impaired T cell priming capacity (Fig. 4, E and F). Cultures with GM-CSF–derived BMDCs revealed a dependence on RALDH for their tolerogenic effect (Fig. 4G). Together, these data show that both murine and human RA-treated DCs rely on AMPK signaling for their tolerogenic potential and suggest that the relative contribution of downstream effector molecules (TGF-β or RALDH) differs depending on the experimental model.

### AMPK controls RA-DCs tolerogenicity independently from metabolic rewiring

**Fig. 5.**
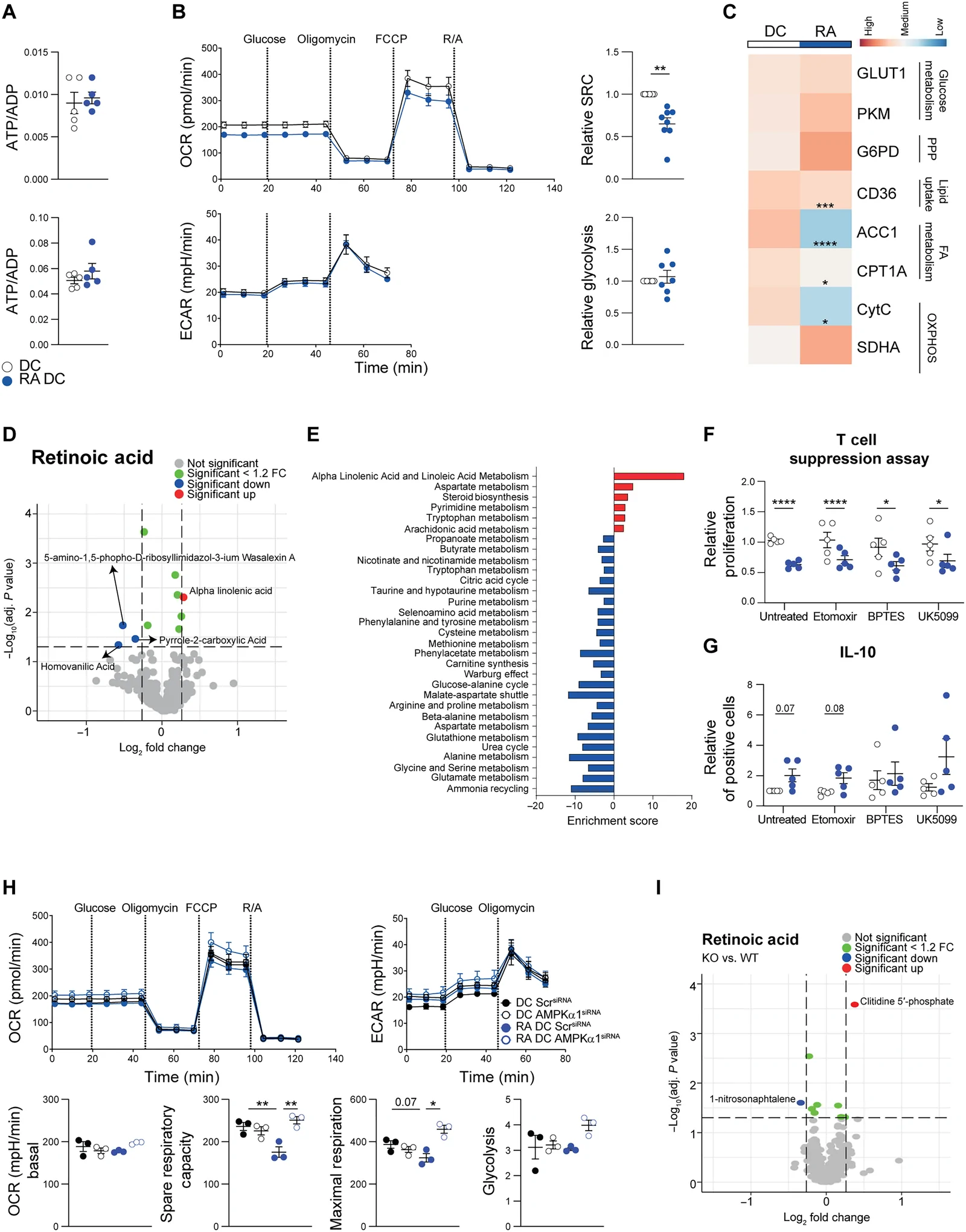
AMPKα1 controls RA-DCs tolerogenicity independently from metabolic rewiring. (**A**) Intracellular levels of AMP, ADP, and ATP in moDCs were measured by HPLC 24 hours after RA stimulation, and the ratio of ADP/ATP and AMP/ATP was quantified. (**B**) Seahorse extracellular flux analysis. Real-time oxygen consumption rate (OCR) (top left) and extracellular acidification rate (ECAR) (bottom left) in moDCs. SRC (top right) and glycolysis (bottom right) were quantified and are displayed as fold change compared to control-stimulated DCs. (**C**) Heatmap of the relative normalized GeoMFI values of intracellular staining for metabolic proteins as determined by flow cytometry. (**D**) Volcano plot showing metabolites differentially modulated in RA-DCs compared to DCs. (**E**) Top modulated metabolic pathways in RA-DCs compared to DCs. (**F** and **G**) moDCs were pre-treated with etomoxir, BPTES, or UK5099 before RA treatment to inhibit FAO, glutaminolysis, or pyruvate oxidation in the mitochondria, respectively, and (F) T cell suppression assay and (G) IL-10 production by T cells were evaluated. (**H** and **I**) moDCs were silenced for AMPKα1 or nontargeting scramble RNA, and 24 hours later, cells were treated with RA. Next, moDCs were treated with LPS, and 24 hours later, they were harvested for extracellular flux analysis or metabolite extraction. (H) Seahorse extracellular flux analysis. Real-time OCR (top left) and ECAR (bottom left) in moDCs. Basal OCR, SRC, basal ECAR, and glycolysis were quantified, shown as fold change compared to scrambled control-stimulated DCs (DC). (I) Volcano plot showing metabolites modulated in RA-DCs AMPKα1^siRNA^ compared to RA-DCs Scr^siRNA^. Data are pooled of four to seven donors [(A) to (I) and (K) to (M)]. Means ± SEM are indicated in the graphs. Paired Student’ *t* test [(A) to (C), (F), and (G)], two-way ANOVA for repeated measurements with Sidák’s posttest (H), or EDGE-R analysis using false discovery rate *P* values [(D) and (I)] were used for the analysis. *P < 0.05, **P < 0.01, ***P < 0.001, and ****P < 0.0001.

In the light of these results, we next aimed to address through which mechanisms AMPK signaling controls the tolerogenic phenotype of RA-DCs. First, to understand how RA may stimulate AMPK signaling in moDCs, we evaluated whether RA treatment could affect cellular energy status by measuring the intracellular levels of AMP, adenosine 5′-diphosphate (ADP), and ATP, as increased ratios in AMP/ATP and ADP/ATP promote AMPKα1 activation (*22*, *23*). However, RA neither altered ATP/ADP nor AMP/ATP ratios, suggesting that RA does not increase AMPK activity through changes in bioenergetic status (Fig. 5A). Correspondingly, minimal RA-induced core metabolic alterations were observed by real-time measurement in glycolysis and OXPHOS rates, with RA only reducing the spare respiratory capacity (SRC) of DCs (Fig. 5B). Likewise, flow cytometry and untargeted metabolomic analyses revealed that RA induced only few metabolic alterations in human DCs, which were largely limited to FA metabolism (Fig. 5, C to E; fig. S4J; and data file S1). In murine Flt3L-induced BM cDC2s, RA induced mitochondrial respiration and glycolysis, of which the former was in part dependent on AMPK (fig. S4K).

We recently reported that pharmacological AMPK activation was sufficient to promote CD103^+^ tolDCs through metabolic reprogramming involving enhanced FAO and glucose oxidation (*26*). However, inhibition of FAO, glutaminolysis, or pyruvate oxidation in the mitochondria with etomoxir, BPTES, or UK5099, respectively, did not compromise the ability of RA-DCs to induce functional T_reg_ cells (Fig. 5, F and G). Moreover, while silencing of AMPKα1 in RA-DCs did restore the loss of SRC induced by RA (Fig. 5H), only few changes in metabolite content were found (Fig. 5I and data file S1). Together, the data suggest that AMPK underpins the RA-induced tolerogenicity of DCs independently from metabolic reprogramming.

### AMPK controls CD103 expression via PGC1α and RALDH activity and TGF-β via FoxO3

**Fig. 6.**
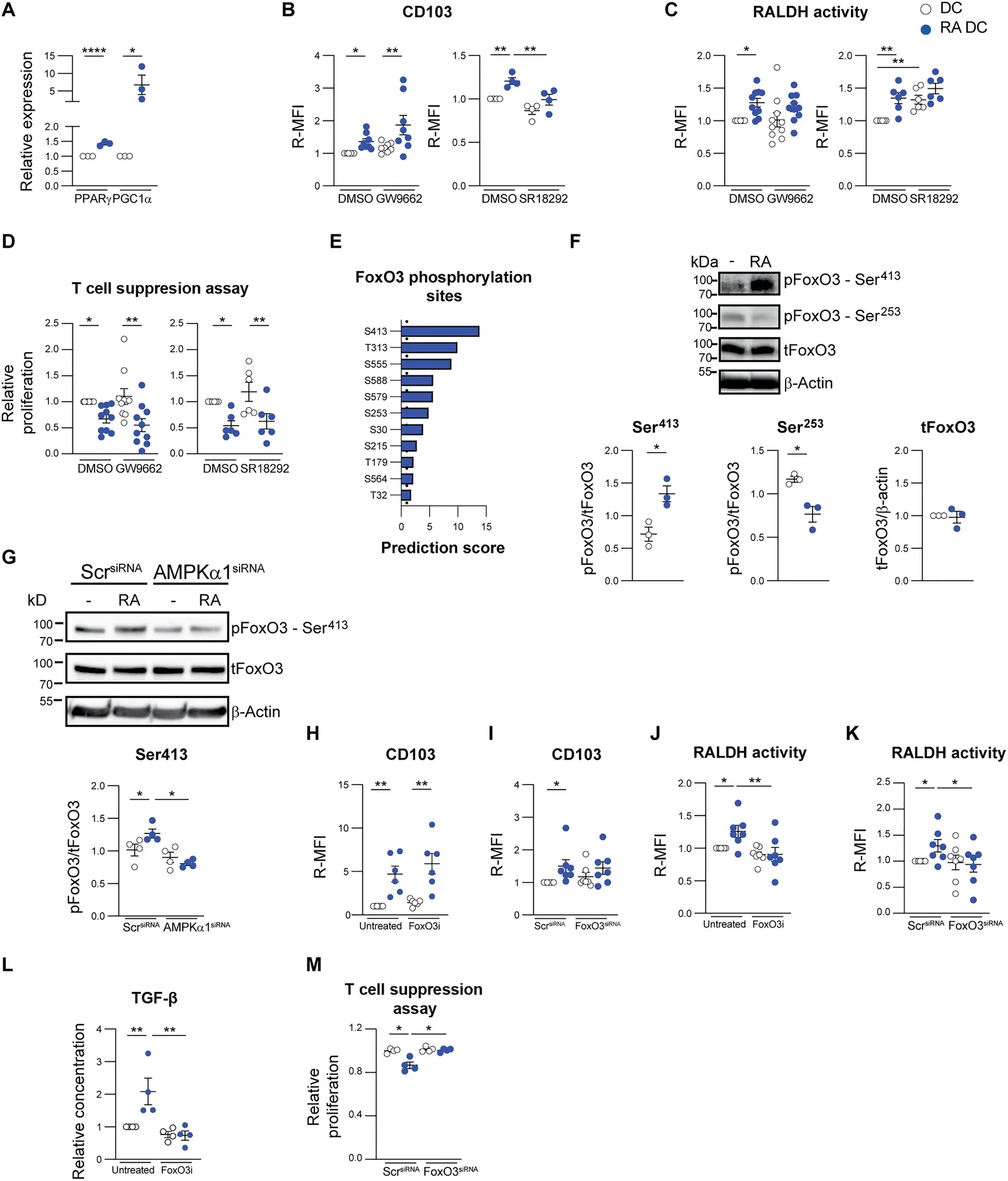
AMPK signals via PGC1α and FoxO3 to control CD103 expression and RALDH activity/TGF-β expression, respectively. (**A**) Gene expression of PPARγ and PGC1α in RA-treated and control moDCs. On day 5 of culture moDCs were treated either with RA or left untreated for 48 hours and mRNA expression levels were determined by reverse transcription quantitative PCR. GAPDH was used as housekeeping gene. (**B** to **D**) On day 5, moDCs were pretreated with either DMSO, PPARγ (GW9662), or PGC1α (SR18292) inhibitors for 30 min before RA treatment, followed by LPS stimulation for 24 hours, and (B) CD103 expression, (C) RALDH activity, and (D) T_reg_ cell inducing capacity were assessed in a T cell suppression assay and quantified by flow cytometry. (**E**) In silico analysis displaying the top predicted AMPK- phosphorylation sites in FoxO3. Dashed line represents the threshold level of AMPK-FoxO3 interaction. (**F**) Representative immunoblot (top) and quantification (bottom) of the expression levels of FoxO3 Ser^413^, FoxO3 Ser^253^, total FoxO3, and β-actin in RA-DCs. (**G**) Representative immunoblot (top) and quantification (bottom) of the expression levels of FoxO3 Ser^413^, total FoxO3, and β-actin in RA-DCs silenced for AMPKα1. (**H** and **I**) Expression levels of CD103 and (**J** and **K**) RALDH activity in RA-DCs silenced for FoxO3 as described in Fig. 3 or treated with a FoxO3 inhibitor. (**L**) Relative TGF-β concentration in supernatant of RA-DC cultures treated with a FoxO3 inhibitor (**M**) moDCs were silenced for FoxO3 as described in Fig. 3, and T cell suppression assay was performed as described in Fig. 2C. Data are a pool of 3 to 10 donors [(A) to (D) and (F) to (L)] or are representative of one of the three independent experiments with four replicates per experiment (M). Statistics are paired Student’s *t* test [(A) and (F)] and Two-way ANOVA for repeated measurements with Sidák’s posttest [(B) to (D) and (G) to (M)] were used to assess statistically significant differences. Means ± SEM are indicated in the graphs. *P < 0.05, **P < 0.01, ***P < 0.001, and ****P < 0.0001.

We therefore set out to explore potential metabolism-independent mechanisms through which AMPKα1 may support the tolerogenic phenotype of human RA-DCs. PGC1α is a substrate of AMPK (*38*) that can act as a coactivator for the nuclear receptor peroxisome proliferator–activated receptor γ (PPARγ) (*39*). As it has been proposed that PGC1α controls RALDH activity in murine CD103^+^ cDC2s (*40*), we wondered whether AMPKα1-driven RALDH activity in RA-DCs is dependent on PPARγ-PGC1α signaling. We found mRNA expression of both PPARγ and PGC1α to be significantly up-regulated in response to RA treatment in DCs (Fig. 6A). However, although pharmacological inhibition of PGC1α, but not PPARγ, did prevent RA-induced expression of CD103 (Fig. 6B), neither inhibition of PGC1α nor PPARγ affected the ability of RA to increase RALDH activity (Fig. 6C) or of RA-DCs to prime T_reg_ cells (Fig. 5D), suggesting that AMPKα1 does not signal through the PPARγ-PGC1α axis to induce RALDH activity and tolerogenicity in RA-DCs. Another transcription factor downstream of AMPK is FoxO3, which belongs to the forkhead-box family of transcription factors and plays a role in a variety of processes including longevity, cell differentiation, and energy homeostasis (*41*). FoxO3 has been shown to serve as positive transcriptional regulator of TGF-β1 (*42*). In silico analysis, using GPS5.0 (*43*) revealed that the top phosphorylated site by AMPK in FoxO3 is S413, which has been shown to promote its transcriptional activity (Fig. 6E) (*44*). Congruently, we found that upon RA treatment, DCs displayed increased phosphorylation at S413 (Fig. 6F). In addition, the phosphorylation of FoxO3 at S253, which is known to prevent its translocation to the nucleus (*45*), was inhibited (Fig. 6F). Silencing AMPKα1 in moDCs abolished the RA-induced phosphorylation of FoxO3 at S413 (Fig. 6G). In addition, while inhibition or small interfering RNA (siRNA)–mediated silencing of FoxO3 in moDCs did not affect expression of surface markers modulated by RA, including CD103 (Fig. 6, H and I), the ability of RA to induce RALDH activity (Fig. 6, J and K),TGF-β production (Fig. 6L) and promote tolerogenic DC function was lost (Fig. 6M). Together, these data show that RA triggers AMPKα1 signaling to promote TGF-β expression, RALDH activity, and CD103 expression via FoxO3 and PGC1α, respectively.

### AMPK signaling in CD11c-expressing cells is dispensable for intestinal T_reg_ cell homeostasis at steady state

Because human and murine RA-DCs lacking AMPKα1 expression were impaired in their ability to prime T_reg_ cells in vitro, we wondered whether in vivo intestinal cDCs from CD11c^ΔAMPKα1^ have a similar defect. We therefore performed unbiased analysis of the CD4^+^ compartment from the siLP, colon, and msLn from both CD11c^WT^ and CD11c^ΔAMPKα1^ mice at steady state (gating strategy shown in fig. S1, F and G). Flow cytometry–based high-dimensional analysis (fig. S5, A and B) and PCA (fig. S5C) could distinguish CD4^+^ T cells from their location based on T cell activation and lineage markers. As expected, a higher proportion of naïve T cells was observed in the msLn, whereas both siLP and colon displayed higher frequencies of CD44^+^ effector T cells, especially in the siLP. A bigger proportion of T_H_17 cells were found in the colon, where most of both Helios^+^ thymus-derived T_reg_ cells (tT_reg_ cells) and RORγt^+^ pT_reg_ cells were also found (fig. S5, A, B, and D). Unbiased analysis revealed 23 phenotypically distinct CD4^+^ T cell clusters within the three tissues (fig. S5, E and F), and few differences in cluster frequencies between naive CD11c^WT^ and CD11c^ΔAMPKα1^ mice were observed (fig. S5, E and G). We found a cluster of Foxp3^−^CD44^+^ antigen-experienced cells to be increased in the siLP of CD11c^ΔAMPKα1^ mice (CD4_08), characterized by increased CTLA4 expression levels (fig. S5, H and I), indicative of recent activation (*46*). Together, the data suggest that, at steady state, absence of AMPK in the CD11c-expressing cell compartment has little effect on intestinal T cell homeostasis, including that of T_reg_ cells.

### AMPK signaling in CD11c-expressing cells is required for up-regulation of the Treg response during *H. polygyrus* infection and constrains antiparasite immunity

**Fig. 7.**
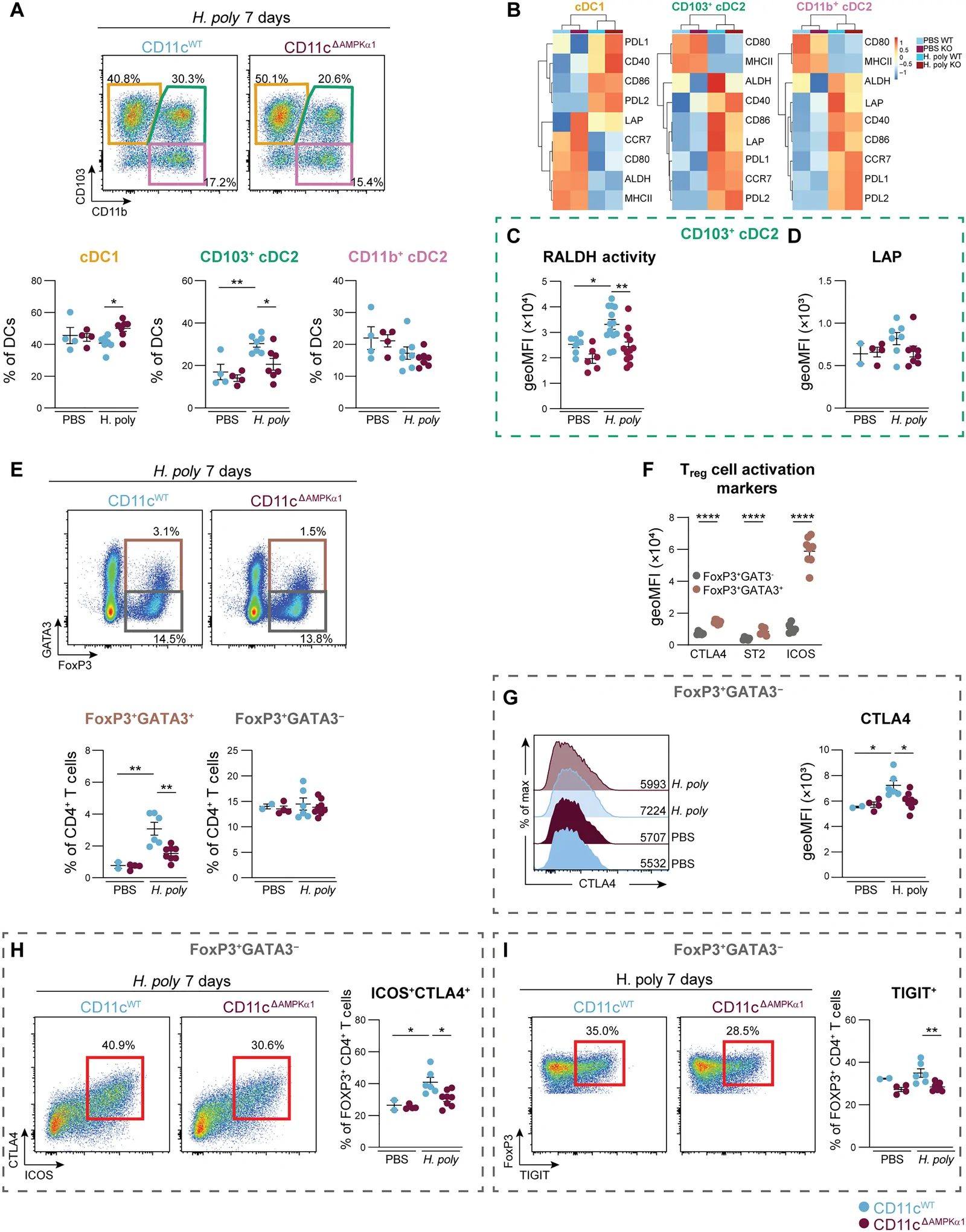
AMPK signaling in CD11c-expressing cells is important for T_reg_ cell induction during *H. polygyrus* infection. Mice were infected with 200 L3 larvae of *H. polygyrus*, and immune cell responses were evaluated in the siLP and draining msLn 7 days postinfection. (**A**) Representative dot plots (top) and quantification of frequency of DCs from siLP of infected CD11c^WT^ and CD11c^ΔAMPKα1^ mice. (**B**) Heatmap displaying relative expression of surface markers in siLP cDC1, CD103^+^ cDC2s, and CD11b^+^ cDC2s from infected CD11c^WT^ and CD11c^ΔAMPKα1^ mice. (**C**) Quantification of RALDH activity in CD103^+^ cDC2s from siLP of infected CD11c^WT^ and CD11c^ΔAMPKα1^ mice. (**D**) Quantification of LAP expression on CD103^+^ cDC2s from msLn of infected CD11c^WT^ and CD11c^ΔAMPKα1^ mice. (**E**) Representative dot plots (top) and quantification of frequency of FoxP3^+^ T_reg_ cell subsets from msLn of infected CD11c^WT^ and CD11c^ΔAMPKα1^ mice. (**F**) Quantification of CTLA4, ST2, and ICOS from GATA3^−^ and GATA3^+^ T_reg_ cells. (**G** to **I**) Quantification of (G) CTLA4, (H) ICOS, and (I) TIGIT from FoxP3^+^GATA3^−^ T_reg_ cells from the msLn of infected CD11c^WT^ and CD11c^ΔAMPKα1^ mice. Data are a representative of one of the two independent experiments with four to eight mice per group. Two-way ANOVA with Sidák’s posttest [(A), (C) to (E), and (G) to (I)] or Student’s *t* test (F) were used to assess statistically significant differences. Means ± SEM are indicated in the graphs. *P < 0.05, **P < 0.01, ***P < 0.001, and ****P < 0.0001. KO, knockout.

Although the T_reg_ cell compartment of CD11c^ΔAMPKα1^ mice appeared largely unaffected during steady state, we wondered whether these mice may have altered T_reg_ cell responses in the context of gut inflammation. To test this, we infected mice with *H. polygyrus*, an enteric murine parasite that elicits a strong type 2 immune response and promotes the expansion of intestinal T_reg_ cells (*47*), which in turn constrain the host’s type 2 immune response, allowing chronic parasite persistence (*48*). We found that the reported expansion of siLP-resident CD103^+^ cDC2s (*49*) around day 7 post *H. polygryus* infection (7DPI) was abrogated in CD11c^ΔAMPKα1^ mice (Fig. 7A). Moreover, we found lower RALDH activity in intestinal CD103^+^ cDC2s from the siLP (Fig. 7, B and C, and fig. S6A) and a trend toward lower LAP expression in CD103^+^ cDC2s from the msLn (Fig. 7D and fig. S6A; *P* = 0.20) in CD11c^ΔAMPKα1^ mice 7DPI, which largely aligns with the in vitro human moDCs data. Recent data have emerged showing an important role for RORγt^+^ DCs in the development of Helios^−^RORγt^+^ pT_reg_ cells (*50*–*52*), but we did not observe differences in the frequency of RORγt^+^ DCs between infected CD11c^WT^ and CD11c^ΔAMPKα1^ mice (fig. S6B).

During the course of *H. polygyrus* infection, the peak of FoxP3^+^ T_reg_ cells occurs around 7DPI in the msLn (*47*). We found a selective increase in frequency of FoxP3^+^ T_reg_ cells expressing GATA3 in the msLn 7DPI (Fig. 7E). These T_reg_ cells were characterized by expression of higher levels of T_reg_ cell effector molecules CTLA4, ST2, and ICOS compared to GATA3^−^ T_reg_ cells (Fig. 7F). The GATA3^+^ T_reg_ cell population was strongly reduced in msLNs of CD11c^ΔAMPKα1^ mice (Fig. 7E). No change was observed in the SiLP at this time point (fig. S6C). In addition, although frequencies of FoxP3^+^GATA3^−^ T_reg_ cells were not different between infected CD11c^WT^ and CD11c^ΔAMPKα1^ mice, the expression of CTLA4 (Fig. 7H), ICOS (Fig. 7I), and TIGIT (Fig. 7J) was significantly reduced on these cells in the latter mice, suggesting that AMPK signaling in cDCs is required for shaping of a proper intestinal T_reg_ cell response during *H. polygryus* infection.

**Fig. 8.**
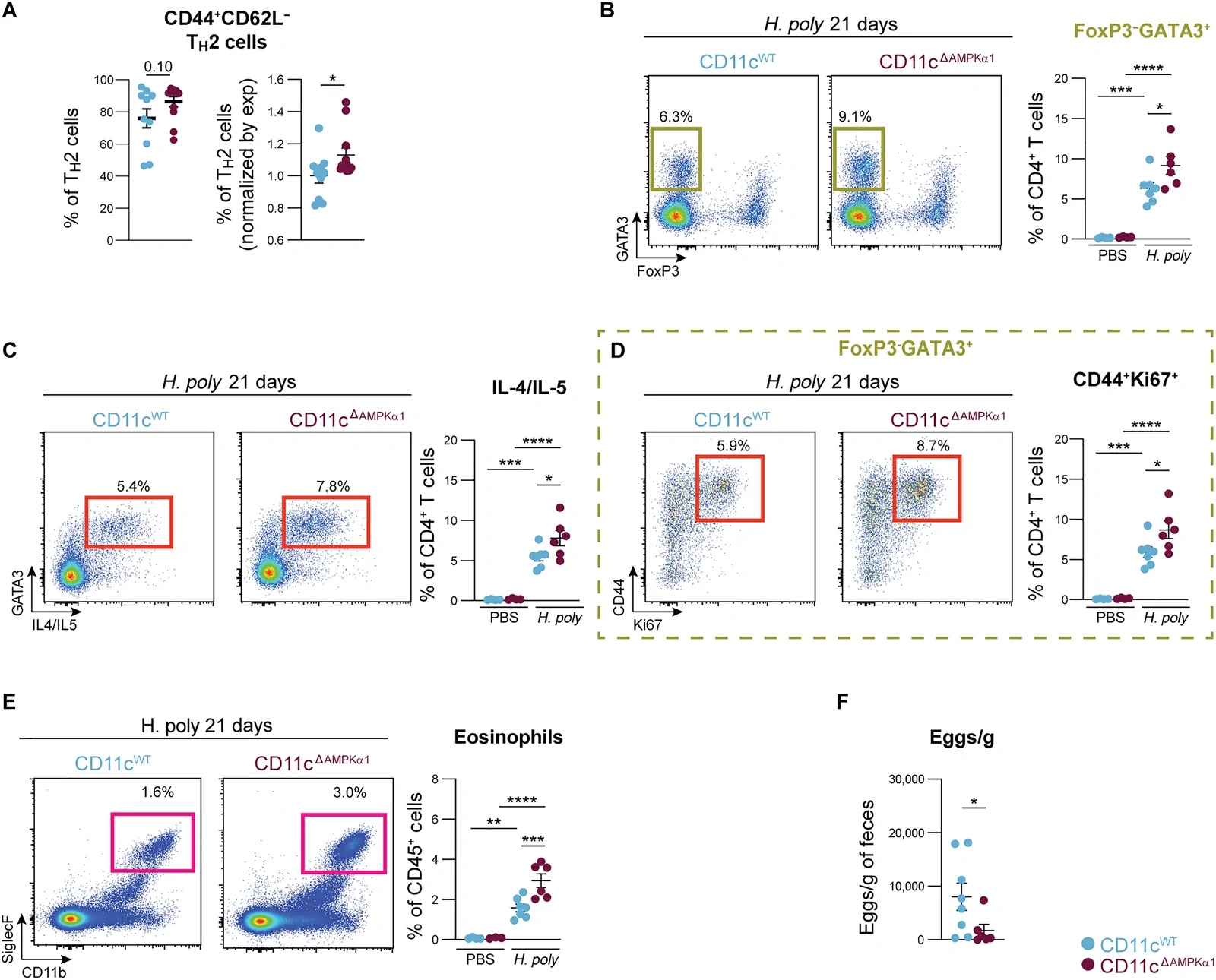
AMPK signaling in CD11c-expressing cells is important to constrain T_H_2 priming during late stages of *H. polygyrus* infection. Mice were infected with 200 L3 larvae of *H. polygyrus* and immune cell responses were evaluated in the draining msLn (A) 7 or [(B) to (F)] 21 days postinfection. (**A**) Frequency of CD4^+^ (left) and normalization per experiment (right) of T_H_2 effector T cells in the mesenteric lymph nodes (msLn) of CD11c^WT^- and CD11c^ΔAMPKα1^-infected mice. Normalization was done by dividing the frequency of T_H_2 effector cells from each mice to the average of WT-infected T_H_2 effector cells calculated per experiment. (**B**) Representative dot plot (left) and frequency of CD4^+^ T cells (right) of FoxP3^−^GATA3^+^ T_H_2 T cells in the msLn of CD11c^WT^ and CD11c^ΔAMPKα1^ infected mice. (**C** to **E**) Frequency of (C) IL-4/IL-5 and (D) effector proliferating CD44^+^Ki67^+^ T_H_2 T cells, and (E) eosinophilia in the msLn of CD11c^WT^- and CD11c^ΔAMPKa^-infected mice. (**F**) Fecal egg counts calculated from the feces of CD11c^WT^ and CD11c^ΔAMPKa^ mice 21 days postinfection with *H. polygyrus*. Data are from two independent experiment with four to seven mice per group (A) or one experiment with four to seven mice per group [(B) to (F)]. Student’s *t* test [(A) and (F)] or two-way ANOVA with Sidák’s posttest [(B) to (E)] or were used to assess statistically significant differences. Means ± SEM are indicated in the graphs. *P < 0.05, **P < 0.01, ***P < 0.001, and ****P < 0.0001.

Given the importance of T_reg_ cells in suppressing type 2 immunity during helminth infections, we next evaluated whether the impairment in T_reg_ cell response observed in msLn of infected CD11c^ΔAMPKα1^ mice would lead to an increased type 2 immune response and may alter resistance to infection. We found effector CD44^+^ T_H_2 cells to be increased 7DPI in the msLn of CD11c^ΔAMPKα1^ mice (Fig. 8A). Also, during the peak of the T_H_2 immune response following *H. polygyrus* infection, around 21DPI (*47*, *53*), an increased accumulation of the GATA3^+^ T_H_2 cells was observed in CD11c^ΔAMPKα1^ mice (Fig. 8B). This aligned with elevated frequencies of IL-4^+^/IL-5^+^ T_H_2 cells (Fig. 8C) and Ki67^+^CD44^+^ proliferating effector T_H_2 cells (Fig. 8D). Frequencies of ST2^+^ precursors T_H_2 cells or type 2 innate lymphoid cells (ILC2s) were not altered in infected CD11c^ΔAMPKα1^ mice (fig. S6D). These immunological changes were correlated with increased eosinophils frequencies in the msLn (Fig. 8E) and a reduction in fecal egg counts in infected CD11c^ΔAMPKα1^ mice (Fig. 8F). Together, the data suggest that AMPK expression in intestinal cDCs is important for their capacity to effectively promote effector function of T_reg_ cells, facilitating parasite fecundity by dampening the host’s T_H_2 immune response.

### AMPK signaling in CD11c-expressing cells is important for T_reg_ cell accumulation and prevention of immunopathology during *S. mansoni* infection

**Fig. 9.**
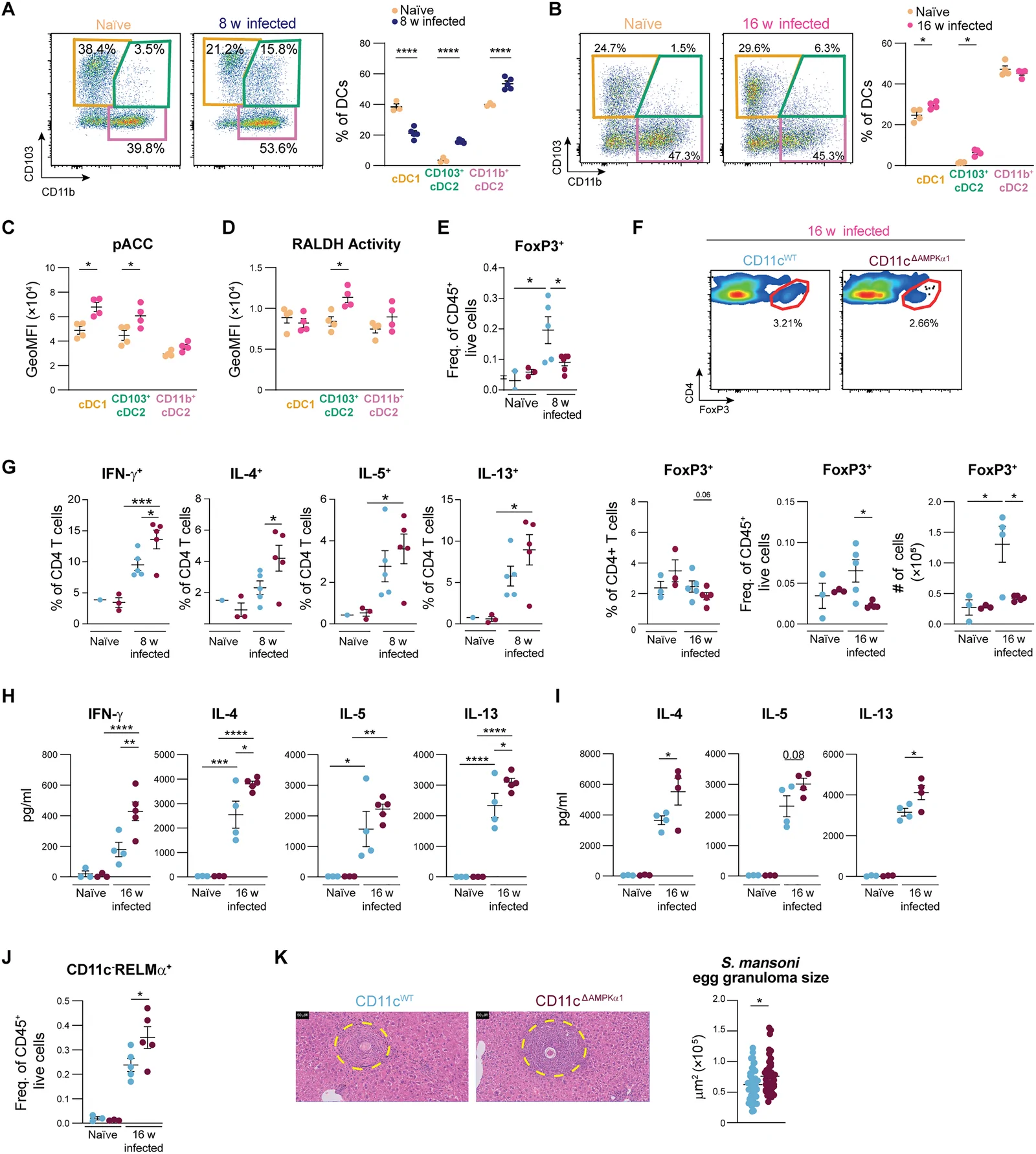
AMPK signaling in CD11c-expressing cells is important for T_reg_ cell induction during *S. mansoni* infection and allergic asthma. Mice were infected with *S. mansoni* and immune cell responses were evaluated in livers and draining hepatic (hLns) and mesenteric lymph nodes (msLns) 8 or 16 weeks later. (**A** and **B**) Representative dot plot (left) and frequency of DCs (right) in the liver of naïve, 8- (A) or 16- (B) week infected WT mice. (**C**) GeoMFI of pACC and (**D**) frequency of RALDH-positive hepatic DC subsets from naïve and 16-week infected WT mice. (**E** and **F**) Representative dot plot staining of FoxP3^+^ T_reg_ cells in the liver of 8- (E) or 16-week (F) *S. mansoni*–infected CD11c^WT^ and CD11c^ΔAMPKα1^ mice. (E) FoxP3^+^ T_reg_ cell frequencies within CD45^+^ live cells in 8 week *S.mansoni* infected mice. (**F**) FoxP3^+^ T_reg_ cell frequencies within CD45^+^ live cells, within CD4^+^ T cells and number in 16-week *S. mansoni*–infected mice. (**G**) Draining hLn cells from 8 week *S. mansoni*–infected mice intracellularly stained for indicated cytokines after polyclonal restimulation with PMA/ionomycin. (**H**) Draining hepatic and (**I**) mesenteric lymph node cells from 16 week *S. mansoni*–infected mice were stimulated with soluble egg antigen (SEA) and analyzed for production of indicated cytokines by cytometric bead assay (CBA). (**J**) Frequency of CD11c^−^RELMα^+^ macrophages in the liver of 16-week *S. mansoni*–infected CD11c^WT^ and CD11c^ΔAMPKα1^ mice. (**K**) Representative hematoxylin and eosin (H&E) staining of liver sections and quantification of size of individual egg granuloma from 16-week infected CD11c^WT^ and CD11c^ΔAMPKα1^ mice. Dashed line marks border of granuloma around the eggs. Scale bars, 50 μm. Data are from two experiments using three to five mice per group [(A) to (J)]. Student’s *t* test [(A) to (D) and (K)] or two-way ANOVA with Sidák’s posttest [(E) to (J)] were used to assess statistically significant differences. Means ± SEM are indicated in the graph. *P < 0.05, **P < 0.01, ***P < 0.001, and ****P < 0.0001.

To extend these findings to a nonenteric helminth infection, we infected mice with parasitic helminth *S. mansoni*, which is one of the most well studied helminth infections known for strong induction of T_reg_ cells (*54*, *55*). During schistosomiasis, major drivers of pathology are the parasitic eggs released by mature worms residing in the mesenteric vasculature that, because of egg-induced type 2 inflammation, lead to granulomatous tissue lesions in the intestine and liver. During this infection, the induction of pT_reg_ cells is crucial for preventing overzealous T_H_2 responses and uncontrolled granuloma formation, especially during the chronic stages of infection (*56*). We observed the appearance of CD103^+^ cDC2s in the liver during acute (8 week) and chronically (16 week) infected mice (Fig. 9, A and B) displaying increased AMPK signaling, as determined by pACC levels (Fig. 9C) and RALDH activity (Fig. 9D). In contrast to the WT littermates, infected CD11c^ΔAMPKα1^ mice failed to increase T_reg_ cell frequencies and numbers in their livers at both time points (Fig. 9, E and F). In addition, an increase in both T_H_1 and T_H_2 cytokine secretion was observed in 8- and 16-week infected CD11c^ΔAMPKα1^ mice in both the hepatic lymph nodes (Fig. 9, G and H) and msLn (Fig. 9I). Consistent with an uncontrolled type 2 immune response, we found a higher frequency of alternatively activated CD11c^−^RELMα^+^ Mphs (Fig. 9J) and an increased liver egg granuloma size in chronically infected CD11c^ΔAMPKα1^ mice compared to infected WT littermates (Fig. 9K), indicative of enhanced immunopathology. Together, the data suggest that AMPK signaling in CD11c-expressing cells is important to induce the differentiation and/or expansion of FoxP3^+^ T_reg_ cells and to restrain excessive effector T helper cell responses and limiting egg-induced granulomatous inflammation during *S. mansoni* infection.

### Intestinal cDC2s from *H. polygyrus*–infected mice, prime FoxP3^+^ T_reg_ cells in an AMPK-dependent manner

**Fig. 10.**
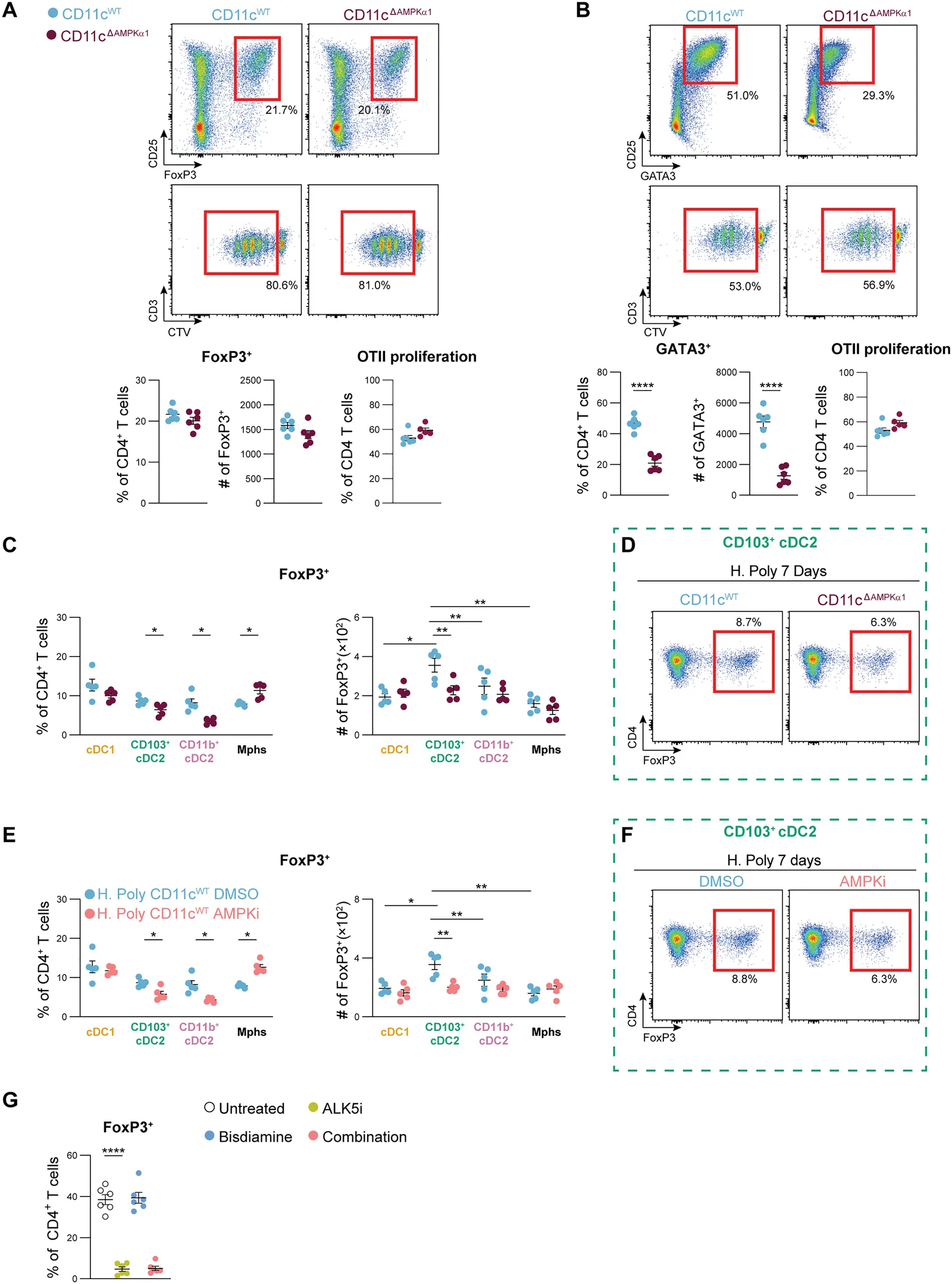
Intestinal cDC2s from *H. polygyrus*–infected mice, prime FoxP3^+^ T_reg_ cells in an AMPK-dependent manner. (**A** and **B**) CD64^−^MHCII^+^CD11c^+^ bulk intestinal DCs were sorted by flow cytometry, pulsed with OVA in the presence of LPS and CpG-B for 2 hrs, and cocultured in a 1:5 ratio with naïve OT-II T cells in the presence of (A) TGF-β for T_reg_ cell or (**B**) IL-4 for T_H_2 DC-priming capacity. In some cocultures, OT-II cells were labeled with CTV to track cell proliferation. (**C** to **G**) Mice were infected with 200 L3 larvae of *H. polygyrus* and DC subsets, and Mphs from siLP were sorted by flow cytometry. Subsequently, cells were pulsed with OVA in the presence of [(C) and (D)] LPS, CpG-B, and [(E) and (F)] AMPKi for 2 hours and cocultured in a 1:5 ratio with naïve OT-II T cells in the presence of TGF-β for T_reg_ cell DC-priming capacity. [(C) and (E)] T_reg_ cell frequency within CD4 T cells (left) and total T cell (middle) and T_reg_ cell numbers (right) in cultures determined 3 days after coculture with different siLP DC subsets and Mphs. (G) T_reg_ cell frequency within CD4^+^ T cells (left) and T_reg_ cell numbers (right) in cultures determined 3 days after coculture with sorted CD103^+^ cDC2 pretreated with RALDH bisdiamine and/or TGF-β signaling inhibitor ALK5. [(D) and (F)] Representative dot plots from T_reg_ cells obtained 3 days after coculture of naïve OT-II cells and CD103^+^ cDC2s. Data are from one experiment using six mice per group [(A) and (B)] or one experiment with five mice per group [(C) to (G)]. Student’s *t* test [(A), (B), and (C)] or two-way ANOVA with Sidák’s posttest [(C) and (E)] was used to assess statistically significant differences. Means ± SEM are indicated in the graph. *P < 0.05, **P < 0.01, and ****P < 0.0001.

Last, we aimed to identify the intestinal cDC subset(s) that rely on AMPK signaling for induction of intestinal T_reg_ cells. First, when bulk CD64^−^MHCII^+^CD11c^+^ siLP cDCs from CD11c^ΔAMPKα1^ mice were cocultured with naïve OT-II T cells for 3 days in the presence of TGF-β to evaluate their cell intrinsic T_reg_ cell priming capacity, no significant reduction in general T cell proliferation or T_reg_ cell priming was found (Fig. 10A). This largely aligns with the absence of major changes in intestinal Treg populations in naïve CD11c^ΔAMPKα1^ mice (fig. S5). Notably, this same in vitro assay in the presence of IL-4 to promote T_H_2 priming revealed that the loss of AMPK signaling in intestinal cDCs significantly compromised their T_H_2 differentiation capacity, without affecting T cell proliferation in this setting (Fig. 10B), suggesting that the increase in T_H_2 response observed during helminth infection is likely secondary to impaired T_reg_ cell priming, rather than a direct inhibitory effect of AMPK on the T_H_2-priming capacity of cDCs.

Next, the assessment of the ability to prime de novo T_reg_ cells by separately sorted cDC1s, CD103^+^ cDC2s, CD11b^+^ cDC2s, and CD11b^+^CD64^+^ Mphs isolated from steady-state intestines did however reveal some dependency on AMPK for T_reg_ cell priming of specific subsets. Specifically, we found that AMPK-deficient cDC1s and, to a lesser extent, Mphs were impaired in their ability to prime T_reg_ cells, as evidenced by reduced accumulation of T_reg_ cells numbers in these cultures (fig. S6, E and F). This defect only pertained to de novo induction of T_reg_ cells, as we did not observe any difference in the ability of these cDCs lacking AMPK to promote proliferation of preexisting T_reg_ cells (fig. S6, G and H). Collectively, these findings suggest that particularly intestinal cDC1s require AMPK for optimal priming of T_reg_ cells ex vivo under steady state, but that this defect is not sufficient to compromise T_reg_ cell priming in situ in the presence of other DC populations as evidenced from the in vitro cultures with bulk cDCs from CD11c^ΔAMPKα1^ mice (Fig. 10A) and in vivo steady-state data showing no major alterations in the T_reg_ cell pool in CD11c^ΔAMPKα1^ mice (fig. S5).

Given the pronounced defect in T_reg_ cell accumulation in vivo in CD11c^ΔAMPKα1^ mice during infection, we hypothesized that specifically during inflammatory settings, intestinal cDCs could become overall more reliant on AMPK for induction of T_reg_ cells. To test this, we sorted intestinal cDCs subsets and Mphs from 7-day *H. polygyrus*–infected mice and cocultured them with naïve OT-II T cells. In this context, intestinal CD103^+^ cDC2s showed the strongest capacity to prime T_reg_ cells (Fig. 10C). CD103^+^ cDC2s and CD11b^+^ cDC2s from infected CD11c^ΔAMPKα1^ mice displayed impaired priming of Foxp3^+^ T_reg_ cells in terms of frequency and, for the former, also in numbers (Fig. 10, C and D). In addition, sorted cDCs from infected CD11c^WT^ mice pretreated with an inhibitor of AMPK (AMPKi) mirrored the results observed from infected CD11c^ΔAMPKα1^ mice (Fig. 10, E and F). Last, we found T_reg_ cell induction by intestinal CD103^+^ cDC2s ex vivo to be primarily dependent on TGF-β signaling (Fig. 10G). Together, this suggests that during *H. polygyrus* infection, AMPK signaling in intestinal cDC2s—particularly in CD103^+^ cDC2s—is crucial for T_reg_ cell induction and that this is mediated by TGF-β.

## DISCUSSION

Despite the well-established function for AMPK signaling in promoting catabolic metabolism (*57*, *58*) and the growing appreciation for catabolic metabolism as central component for the tolerogenic functions of cDCs (*2*, *20*), the importance of AMPK for tolDC function in vivo has not yet been addressed. Here, we provide evidence that AMPK signaling is crucial for supporting the tolerogenic properties of RA-tolerized CD103^+^ DCs in vitro and demonstrate that in vivo AMPK signaling, particularly in CD103^+^ cDC2s, is crucially important for intestinal T_reg_ cell accumulation during different inflammatory settings.

We found that in naïve mice, intestinal cDCs display higher levels of AMPK signaling compared to cDCs from other tissues and msLN and increased expression of proteins linked to a catabolic profile, particularly in CD103^+^ cDC2s. Unexpectedly, despite the well-described role of AMPK signaling in controlling catabolic metabolism, we did not observe major differences in the metabolism of intestinal cDCs from CD11c^ΔAMPKα1^ mice when compared to their WT littermates. This was also true for our in vitro model using human RA-treated DCs mimicking intestinal CD103^+^ tolerogenic DCs that were silenced for AMPKα1, suggesting a dissociation from metabolic rewiring and AMPK activation in these tolerogenic DCs. This is different from our recent work, showing that an allosteric AMPK activator induces tolerogenic human CD103^+^ moDCs through rewiring of glucose and fatty acid metabolism (*26*). We hypothesize this to be due to a difference in AMPK activation strength and duration; relatively mild AMPK activation by RA versus a potent and sustained AMPK activation by a direct allosteric AMPK activator, which in the latter case additionally results in substantial metabolic reprogramming, which may become the dominant driver behind the tolerogenic phenotype of the DCs. On the other hand, in the context of RA treatment, with weaker AMPK activation and consequently less metabolic reprogramming, metabolism-independent pathways (*59*) may become more important for AMPK-driven control of the tolerogenic properties of DCs, such as those involving PGC1α and FoxO3, as we find in this study. In addition, AMPK activation is known to suppress nuclear factor κB (NF-κB) signaling (*60*), of which alternative NF-κB family member RelB was recently shown to limit the tolerogenic potential of intestinal DCs (*61*). Although a direct effect of AMPK signaling on RelB activity has not been reported, it would be interesting to explore whether there is a functional link between RelB signaling and AMPK-dependent tolerogenic responses by DCs in the gut.

In our in vitro human RA-DC model, we found that RA treatment stimulated AMPK activation and that hallmark characteristics of these cells, increased CD103 expression, RALDH activity, and TGF-β were crucially dependent on AMPK signaling. While AMPK-driven CD103 expression was dependent on PGC1α, we provide evidence for a central role for FoxO3 in mediating AMPK-driven RALDH activity and TGF-β secretion following stimulation with RA. This is largely consistent with an earlier study showing an important role for FoxO3 in dampening DC immunogenicity (*62*, *63*). Functionally, increased RALDH activity in murine RA-GMDCs was critical for tolerogenic function in vitro. This was not the case for human RA-DCs or CD103^+^ cDC2s, which appeared to rely on TGF-β signaling, highlighting both commonalities and DC model-specific differences through which the RA-AMPK signaling axis controls DC tolerance. The most well-studied mode of action through which RA is known to alter cell biology, including the induction of tolerogenic features of DCs, is through the activation of its nuclear receptor RAR (*64*–*66*). If and how RA-driven AMPK signaling converges with RAR signaling to control expression of tolerogenic genes remains an open question that warrants further investigation.

Several studies have linked RA to AMPK activation (*67*–*69*), although underlying mechanisms have been poorly defined. In cancer cell lines, an RA-induced reduction in ATP levels has been suggested to promote AMPK activation (*70*); however, we found no changes in ADP/ATP ratios following RA treatment, pointing toward a different mechanism of activation. A recent study revealed that RA may potentially directly interact with AMPK to promote its activity (*71*), and it is tempting to speculate that this may in fact be the key mode of action through which RA controls AMPK activity in DCs. Given these in vitro findings and the observation of a strong AMPK activation in cDCs from the intestine that is rich in RA, it is conceivable that also in the mucosal microenvironment RA is a major driver of AMPK activation in cDCs. Nonetheless, the contribution of other gut-derived factors to promote AMPK activation in mucosal cDCs cannot be ruled out. For instance, gut microbiota–derived products such as the short chain fatty acids (SCFAs) butyrate, acetate, and propionate have been shown to induce AMPK activation in other cell types (*72*–*74*). Further studies will be needed to define the relative contribution of these local cues to AMPK activation.

The in vitro findings linking AMPK signaling to RALDH activity and CD103 expression were corroborated in vivo, as CD11c^ΔAMPKα1^ mice harbored reduced RALDH activity and CD103 expression in intestinal cDC2s. The observed reduced frequency of CD103^+^ cDC2s was accompanied by a trend toward a concomitant higher frequency of CD11b^+^ cDC2s in these mice, suggesting loss of CD103 expression, rather than cell death of these cDC2s due to loss of AMPK signaling. This suggests a conserved AMPK-dependent mechanism not limited to a particular cDC subset but perhaps relevant to tissue-resident cDCs involved in mucosal tolerance. RALDH activity in intestinal CD103^+^ cDC2s and lung cDC1s has been linked to the ability of these cells to induce T_reg_ cell responses (*75*–*78*). Nonetheless, CD11c^ΔAMPKα1^ mice did not show clear defects in the intestinal T_reg_ cell pool under steady-state conditions. This may suggest that the reduction in RALDH activity and/or TGF-β expression as a consequence of AMPKα1 loss in the cDC compartment during steady state is not sufficient to affect pT_reg_ cell differentiation and maintenance. (*79*, *80*). A potential additional explanation for a largely intact intestinal T_reg_ cell compartment in CD11c^ΔAMPKα1^ mice may come from recent reports that have identified a subset of intestinal RORγt^+^ DCs, distinct from cDCs, which is critical for neonatal pT_reg_ cell differentiation and seeding in mice (*50*–*52*, *81*) and which we found to be present in equal numbers in CD11c^ΔAMPKα1^ mice. However, it is still not fully elucidated to what extent these cells contribute to shape the adult pTreg pool in the gut (*79*).

Although we did not find significant changes in T_reg_ cell populations as a consequence of AMPKα1 deficiency in cDCs at steady state, these mice did show a clear defect in effector T_reg_ cell accumulation in the context of *H. polygyrus* and *S. mansoni* infection. This suggests that AMPK signaling in cDCs is required for either T_reg_ cell differentiation and/or expansion during an immunological challenge. Our ex vivo data suggest that the loss of AMPK in CD103^+^ cDC2s primarily compromises T_reg_ cell priming from naïve CD4^+^ T cells and not the expansion of preexisting T_reg_ cells. During the peak of the T_reg_ cell response during *H. polygyrus* infection (7DPI) (*47*), we found a population of GATA3^+^FoxP3^+^ T_reg_ cells to accumulate, characterized by a strong immunoregulatory phenotype that was selectively absent in infected CD11c^ΔAMPKα1^ mice. T_reg_ cells lacking GATA3 expression have been shown to have impaired suppressive function (*82*), and the absence of GATA3^+^ T_reg_ cells has been linked to increased inflammation across various inflammatory conditions (*82*–*84*). The fact that the total T_reg_ cell numbers did not differ between WT and conditional knockout mice, points toward a crucial role for AMPK signaling in cDCs in supporting the activation of highly active T_reg_ cells, rather than being important for de novo T_reg_ cell differentiation in this disease setting. In both *H. polygyrus* and *S. mansoni* infections, the reduction in T_reg_ cell accumulation was associated with an increased type 2 immune response. Consistent with an earlier study (*27*), we found that loss of AMPK signaling in intestinal cDCs impaired their ability to support T_H_2 priming, suggesting that the increase in T_H_2 response observed during helminth infection is secondary to impaired T_reg_ cell priming, as reported before (*54*), rather than AMPK deficiency endowing intestinal cDCs with an intrinsic increased capacity to prime a T_H_2 response. This potentiated type 2 immune response resulted in increased protective immunity to *H. polygyrus* infection as evidenced by decreased fecal egg count but worsened pathology in chronic *S. mansoni* infection reflected by an increased granuloma size around liver eggs. Apart from an increased T_H_2 response, we also observed an increase in T_H_1 polarization in *S. mansoni* infected CD11c^ΔAMPKα1^ mice. To what extent this may be secondary to an impaired T_reg_ cell response or due to a cell-intrinsic increased potential of AMPK-deficient cDC1s to promote this response remains to be determined.

In contrast to the data from naïve mice, we found that CD103^+^ cDC2s isolated from the siLP of *H. polygyrus*–infected mice were most efficient in inducing T_reg_ cells, a capacity that was dependent on AMPK signaling. The signals during *H. polygyrus* infection that led to this shift toward reliance on AMPK signaling in cDC2s for promoting T_reg_ cell function remain to be determined, but given that helminth-derived antigens are preferentially sensed by cDC2s (*85*–*87*) and can directly activate AMPK (*88*) makes them likely candidates. We found that *H. polygyrus* infection increased both RALDH activity and LAP expression in an AMPK-dependent manner in CD103^+^ cDC2s and that specifically TGF-β signaling was required for their T_reg_ cell priming capacity. This aligns with our findings with in vitro RA-treated human DCs and together uncovers AMPK-FoxO3-TGF-β as a key signaling axis supporting intestinal tolerogenic responses by DCs in the gut. Last, the in vitro pharmacological inhibition of AMPK signaling in siLP cDC2s from *H. polygyrus*–infected CD11c^WT^ mice phenocopied the defects in T_reg_ cell induction observed in cDC2s from CD11c^ΔAMPKα1^ mice. This highlights the feasibility and potential of acute pharmacological targeting of this pathway to control tolerogenic responses.

This study has several limitations. First, although our in vitro data show that RA can activate AMPK signaling, we do not know to what extent RA is required for AMPK activation and AMPK-dependent tolerization of intestinal cDCs in vivo. In this context, it is relevant to note that we found that in some of our experiments, the effect of loss of AMPK signaling on T_reg_ cell induction was also evident in the absence of RA stimulation, indicating that the importance of AMPK in T_reg_ cell induction by DCs may not be limited to RA-driven tolerance. Second, in vivo T_reg_ cell depletion studies in combination with adoptive transfer of antigen-specific naïve T cells would be needed to formally establish to what extent the observed increase in T_H_1 and T_H_2 response in helminth-infected CD11c^ΔAMPKα1^ mice is secondary to an impaired T_reg_ cell response or due to a cell-intrinsic increased potential of DCs to prime these responses. Last, the relative contribution of TGF-β signaling and RALDH activity to AMPK-driven tolerance induction by DCs in vivo remains to be established.

In conclusion, our work has revealed AMPK signaling as a crucial pathway in controlling the homeostasis of intestinal CD103^+^ cDC2s and for induction of T_reg_ cell responses during immunological challenge. Our findings may help with the rational design of new therapeutic approaches that target this signaling axis in DCs to control inflammatory responses.

## MATERIALS AND METHODS

### Mice

Itgax (CD11c)-cre mice (*89*) were crossed to Prkaa1 (AMPKα1)-flox mice (*90*). For experiments, AMPKα1-deficient Itgax^cre/wt^ × Prkaa1^fl/fl^ mice were compared to AMPKα1-sufficient Itgax^wt/wt^ × Prkaa1^fl/fl^ littermates. These mice, OVA-specific CD4^+^ T cell–receptor transgenic mice (OT-II) and WT mice, both male and female and all on a C57BL/6J background, were bred under specific pathogen–free conditions at the Leiden University Medical Center (LUMC), Leiden, Netherlands. Mice were culled through cervical dislocation. Anesthesia with ketamine with either dexdomitor or xylazine was used for *S. mansoni* infection. Animal experiments were performed when the mice were between 8 and 16 weeks old. Animal experiments were performed in accordance with local government regulations, EU Directive 2010/63EU and Recommendation 2007/526/EC, regarding the protection of animals used for experimental and other scientific purposes, as well as approved by the Dutch Central Authority for Scientific Procedures on Animals (CCD) under animal license number AVD116002015253.

### Differentiation of moDCs

Monocyte-derived DCs (moDCs) were differentiated from monocytes that were isolated from buffy coats from healthy volunteers that donated blood to the National Blood Bank Sanquin (Amsterdam, Netherlands) with informed consent. In brief, blood was diluted 1:2 in Hanks’ balanced salt solution (HBSS) and placed on 12 ml of Ficoll to obtain mononuclear cells. The solution was centrifuged at 600*g* for 30 min at 18°C, without braking. Subsequently, the formed mononuclear cell layer was removed and placed in a new tube containing HBSS for further centrifugation at 1000 rpm for 20 min. The cells were washed two more times at 1000 rpm for 20 min, and monocytes were isolated using CD14 MACS beads (Miltenyi) according to the manufacturer’s recommendations, routinely resulting in a monocyte purity of >95%. Subsequently, 2 × 10^6^ per well of monocytes were cultured in six-well plate in RPMI medium supplemented with 10% fetal calf serum (FCS), penicillin (100 U/ml), streptomycin (100 μg/ml), 2 mM glutamine, human rGM-CSF (10 ng/ml; Invitrogen), and human rIL-4 (0.86 ng/ml; R&D Systems) to differentiate them into moDCs. On day 2/3 of the differentiation process, medium was refreshed, and the same concentration of cytokines was added to the cells. On day 5, immature DCs were either treated with 1 μM RA (RA DCs) or left untreated (mDCs). On day 6, immature DCs were stimulated with ultrapure lipopolysaccharide (LPS) (100 ng/ml; *Escherichia coli* 0111 B4 strain, InvivoGen, San Diego). In some experiments, at day 5, moDCs were treated with 10 μM ALK5 inhibitor (TGF-β RI kinase inhibitor, Sigma Technologies), 10 μM PPARγ inhibitor (GW9662), 80 μM FoxO3 inhibitor carbenoxolone (CBX), AMPK inhibitor (BAY-3827, #HY-112083, MedChemExpress), and 20 μM PGC1α inhibitor (SR18292) for 30 min to 1 hour before treatment with RA.

### Extracellular flux analysis

The metabolic characteristics of moDCs and FLT3-DCs were analyzed using a Seahorse XFe96 Extracellular Flux Analyzer (Seahorse Bioscience). In brief, 5 × 10^4^ moDCs or FLT3-DCs were plated in unbuffered, glucose-free RPMI supplemented with 5% FCS and left to rest 1 hour at 37°C before the assay in an CO_2_-free incubator. Subsequently, extracellular acidification rate and oxygen consumption rate were analyzed in response to glucose (10 mM; port A), oligomycin (1 μM for moDCs and 2 μM for FLT3-DCs; port B), fluoro-carbonyl cyanide phenylhydrazone (FCCP) (3 μM for moDCs and 1.5 μM for FLT3-DCs; port C), and rotenone/antimycin A (1/1 μM for moDCs and 1.5/1.5 μM for FLT3-DCs; port D) (all Sigma-Aldrich). In some experiments, LPS was added in port B after glucose injection.

### RALDH activity

RALDH activity assay was performed with Aldefluor kit (STEMCELL Technologies) according to the manufacturer’s protocol. The RALDH activity assay is a flow cytometry–based assay in which fluorescent signal is directly proportional to enzymatic activity of RALDH. Briefly, cells were harvested, transferred to a V bottom 96 well plate, washed in phosphate-buffered saline (PBS), stained with the Fixable Aqua Dead Cell Stain Kit (Invitrogen) for 15 min at room temperature (RT), and then resuspended in 200 μl of assay buffer. A 3 ml (end concentration of 45 μM) of RALDH inhibitor diethylaminobenzaldehyde (DEAB) was added to an empty well. Next, 1 μl (1.83 μM) of Aldefluor reagent was added to the well with the cells and immediately homogenized and transferred to the well containing DEAB. Samples were incubated for 30 min at 37° C and kept in assay buffer until the measurement in a BD FACS Canto. For the in vivo experiments, samples were stained with 146 nM of Aldefluor followed by staining with mix of antibodies diluted in assay buffer. Cells were kept in assay buffer until the measurement in a five laser Cytek Aurora.

### SCENITH assay

A total of 5 × 10^5^ intestinal cells were plated in a 96-well untreated V-bottom plate, washed with PBS, and plated in 90 μl of methionine-free media (Sigma-Aldrich, R7513), 1× GlutaMAX, and 10% dialyzed FCS. Cells were methionine starved for 15 min before addition of 10 μl of indicated inhibitor(s) [media, 2 μM oligomycin, and 100 mM 2DG (Sigma-Aldrich, D8375)] and subsequently incubated another 15 min. Homopropargylglycine (Click Chemistry Tools, 1067) was added at a final concentration of 100 μM and incubated for 30 min before being washed 2× with cold PBS, live/dead stain and fixed with 2% ultrapure paraformaldehyde for 15 min. Next, cells were permeabilized with PBS containing 0.1% Saponin for 15 min and washed 2× in PBS before the addition of Click reaction mix. The reaction mix was made by sequential addition of 10 mM Sodium Ascorbate (Sigma-Aldrich, A7631), 1 mM THPTA (Click Chemistry Tools, 1010), 10 mM aminoguanidine (Cayman, 81530), 0.5 μM AF647plus-azide (Vectorlabs CCT-1482), and 1× PBS to CuSO_4_ [1 mM final concentration, (Sigma-Aldrich, 209198)]. Samples were incubated for 30 min in the dark at room temperature. Cells were washed with fluorescence-activated cell sorting buffer, and surface staining was performed as normal.

### Metabolomics analysis

Metabolites were extracted from 5.0 × 10^5^ moDCs. Cells were washed with 75 mM ammonium carbonate (A9516, Sigma-Aldrich) in high-performance liquid chromatography (HPLC)–grade water (15651400, Honeywell) at 37°C, before metabolite extraction for 3 min in 600 μl of 70% ethanol at 70°C. Samples were centrifuged at 14,000 rpm for 10 min at 4°C, and supernatants were shipped to General Metabolics for analysis as previously described (*91*). Briefly, polar metabolites were analyzed on a nontargeted metabolomics platform in negative ion mode. A total of 886 ions were identified, of which 632 were annotated according to the human Kyoto Encyclopedia of Genes and Genomes (KEGG) database. Data filtering based on interquartile range, log transformation, and normalization (mean-centered and divided by the standard deviation) was done using Metaboanalyst (www.metaboanalyst.ca), as was further analysis including pathway enrichment and differential expression analysis.

### Human T cell culture and analysis of T cell polarization

For analysis of T cell polarization, moDCs were cultured with allogenic naive CD4^+^ T cells for 7 days in the presence of staphylococcal enterotoxin B (10 pg/ml). On day 7, cells were harvested and transferred to a 24-well plate and cultured in the presence of rhuIL-2 (10 U/mL; R&D Systems) to expand the T cells. Two days later, 2× concentrated rhuIL-2 was added to the well, and cells were divided into two wells. Then, two to three days later, intracellular cytokine production was analyzed after restimulation with phorbol 12-myristate 13-acetate (PMA; 100 ng/ml) and ionomycin (1 μg/ml; both Sigma-Aldrich) for a total of 6 hours; brefeldin A (10 μg/ml; Sigma-Aldrich) was added during the last 4 hours. Subsequently, the cells were stained with the Fixable Aqua Dead Cell Stain Kit (Invitrogen) for 15 min at RT, fixed with 1.9% formaldehyde (Sigma-Aldrich), permeabilized with permeabilization buffer (eBioscience), and stained with anti–IL-4, anti–IL-13, anti–IL-10, anti–IL-17A, and anti–IFN-γ for 20 min at 4°C.

### T cell suppression assay

For the analysis of suppression of proliferation of bystander T cells by test T cells, 5 × 10^4^ moDCs were cocultured with 5 × 10^5^ human naive CD4^+^ T cells for 6 days. These T cells (test T cells) were harvested, washed, counted, stained with the cell cycle tracking dye 1 μM CellTrace Violet (#C34571 A, Thermo Fisher Scientific), and irradiated (3000 RAD) to prevent expansion. Bystander target T cells (responder T cells), which were allogeneic memory T cells from the same donor as the test T cells, were labeled with 0.5 μM cell tracking dye carboxyfluorescein diacetate succinimidyl ester (CFSE). Subsequently, 5 × 10^4^ test T cells, 2.5 × 10^4^ responder T cells, and 1 × 10^3^ allogeneic LPS-stimulated DCs were cocultured for additional 6 days. Proliferation was determined by flow cytometry, by costaining with CD3, CD4, and CD25 allophycocyanin (APC).

### Generation of bone marrow–derived GM-DCs and FLT3-DCs

BM cells were flushed from mouse femurs and tibia and plated in Nunc Cell-Culture Treated 6-well plate wells (#140675, Thermo Fisher Scientific) at a seeding density of 2 × 10^6^ cells in a volume of 3 ml of complete RPMI [RPMI-1640 supplemented with GlutaMAX and 5% FCS, 25 nM β-mercaptoethanol, penicillin (100 U/ml), and streptomycin (100 μg/ml)], to which recombinant GM-CSF (20 ng/ml; #315-03, PeproTech, Hamburg, Germany) was added for generation of GM-DCs. For FLT3-DCs, BM cells were plated at a density of 5 × 10^6^ cells in a volume of 5mL of complete RPMI to which recombinant FLT3L (150 ng/ml; #130-097-372, Miltenyi Biotec, Bergisch Gladbach, Germany) was added. DCs were differentiated for 8 days (GM-DCs) or 9 days (FLT3-DCs) with media refreshment on day 4. At day 6 (GM-DCs) or day 7 (FLT3-DCs), DCs were either treated with 1 μM RA (Sigma-Aldrich) or left untreated. At day 7, DCs were either left untreated or activated with LPS (100 ng/ml; ultrapure, InvivoGen), and after 24 hours, semi-adherent cells were harvested for various assays.

### In vitro murine T cell priming assay

OT-II cells were negatively isolated with the CD4^+^ T cell Isolation Kit (Miltenyi). 1 × 10^4^ GM or Flt3L-DCs were settled in a round bottom 96-well plate, pulsed with OVA (100 μg/ml; InvivoGen) in combination with LPS for 5 hours, and washed before adding 1 × 10^5^ CTV-labeled OT-II cells in 200 μl of medium. After 4 days, cells were harvested and analyzed for proliferation by assessing CTV dilution. For cytokine production, cells were left for 6 days and then 100 μl of media was removed and replaced with 100 μl of media containing PMA/ionomycin (both from Sigma-Aldrich) in the presence of brefeldin A (Sigma-Aldrich) for 4 hours. Then, cells were stained with Fixable Aqua Dead Cell Stain Kit (Invitrogen) for 15 min at RT, fixed with FoxP3/Transcription Factor Staining Buffer Set (Invitrogen, for FoxP3 detection) for 1 hour at 4°C, permeabilized with permeabilization buffer (eBioscience), and stained for anti-CD3, anti-CD4, anti–IFN-γ, anti–IL-10, anti–IL-17A, anti-FoxP3, and anti-CD25.

### In vitro murine T_reg_ cell proliferation assay

A total of 5.0 × 10^4^ flow cytometry–sorted intestinal cDCs subsets were settled in a round bottom 96-well plate, pulsed with OVA (100 μg/ml; InvivoGen) in combination with LPS (100 ng/ml) and CpG-B (5 μg/ml) for 2 h and washed. CD8^−^CD4^+^CD25^+^ OT-II T cells were sorted from the spleen of naive mice, labeled with 0.5 μM CFSE, and labeled OT-II cells (2.5 × 10^5^) were added together with the cDCs in 200 μl of medium containing TGF-β (0.5 ng/ml) and murine IL-2 (5 ng/ml; BioLegend). After 3 days, cells were harvested and stained with Zombie NIR (BioLegend) for 15 min at RT, fixed with FoxP3/Transcription Factor Staining Buffer Set (Invitrogen) for 1 hour at 4°C, permeabilized with permeabilization buffer (eBioscience), and stained with flow cytometry antibodies.

### In vitro murine T_reg_ cell and T_H_2 priming assays

Naïve OT-II T cells were negatively isolated with the CD4^+^ T cell Isolation Kit (Miltenyi) and where indicated labeled with 0.5 μM CTV. Sorted splenic cDC2s (5.0 × 10^4^), bulk intestinal cDCs, or intestinal cDCs subsets were settled in a round-bottom 96-well plate, pulsed with OVA (100 μg/ml; InvivoGen) in combination with LPS (100 ng/ml), CpG-B (5 μg/ml), and where indicated with AMPK inhibitor (5 mM; BAY-3827, Merck Life Science NV), 10 μM ALK5 inhibitor (TGFb-RI Kinase Inhibitor Sigma Technologies), or 45 μM bisdiamine (WIN 18446, 4736/50; Bio-Techne). After 2 hours, cDCs were washed with cRPMI, and OT-II T cells (5.0 × 10^5^) were added together with the cDCs in 200 μl of medium containing TGF-β (0.5 ng/ml) and murine IL-2 (5 ng/ml; BioLegend) for T_reg_ cell induction or murine IL-4 (20 ng/ml; BioLegend) and murine IL-2 (5 ng/ml; BioLegend) for T_H_2 induction. After 3 days, cells were harvested and stained with Zombie NIR (BioLegend) for 15 min at RT, fixed with FoxP3/Transcription Factor Staining Buffer Set (Invitrogen) for 1 hour at 4°C, permeabilized with permeabilization buffer (eBioscience), and stained with flow cytometry antibodies. In some experiments, OT-II T cells were stained with 1 μM CellTrace Violet to assess proliferation.

### Flow cytometry

In general, single-cell suspensions underwent viability staining for 20 min at RT using the LIVE/DEAD Fixable Aqua Dead Cell Stain Kit (#L34957, Thermo Fisher Scientific; 1:400 in PBS from LUMC pharmacy; Braun, Zeist, Netherlands) or LIVE/DEAD Fixable Blue Cells Stain Kit (#L34957, Thermo Fisher Scientific; 1:1000 in PBS) or Zombie NIR™ (Biolegend, 1:1000 in PBS) and fixation for 15 min at RT using 1.85% formaldehyde (F1635, Sigma-Aldrich) in PBS solution before surface staining with antibodies in PBS supplemented with 0.5% bovine serum albumin (fraction V, #10735086001, Roche, Woerden, Netherlands) and 2 mM EDTA for 30 min at 4°C. Used antibodies are listed in table S1. For detection of metabolic proteins, cells were permeabilized with eBioscience permeabilization buffer (#00-8333-56, Thermo Fisher Scientific), followed by intracellular staining with a cocktail of antibodies against the metabolic proteins. Antibodies were purchased from Abcam and conjugated in house with Abcam Lightening-Link Conjugation kit. See table S2 for further information on metabolic antibodies and fluorochrome conjugation. For polyclonal restimulation, single-cell suspensions were stimulated with both PMA (0.1 μg/ml; #P-8139, Sigma-Aldrich) and ionomycin (1 μg/ml; #I-0634, Sigma-Aldrich) in the presence of brefeldinA (10 μg/ml; all Sigma-Aldrich) for 4 hours. These single-cell suspensions underwent intracellular cytokine staining (ICS) with antibodies in 1x eBioscience Permeabilization Buffer (ebioscience, #00-8333-56).

For the detection of phosphorylated ACC on Ser^79^, cells were prestained with the LIVE/DEAD Blue Dead Cell Stain Kit for 20 min at RT, and after washing, single-cell suspension was returned to a cell incubator (37°C and 5% CO_2_) for 1 hour in cRPMI and fixed with 4% ultrapure formaldehyde (#18814-20, Polysciences, Hirschberg an der Bergstraße, Germany) for 15 min at RT. Single-cell suspensions were first stained with anti-phosphorylated ACC in 1× permeabilization buffer (#00-8333-56, Thermo Fisher Scientific) for 1 hour at room before staining with cocktail antibodies in 1× permeabilization buffer for 30 min at 4°C.

### High-dimensional spectral flow cytometry analysis

Samples were imported in OMIQ software, and parameters were scaled using a conversion factor ranging from 6000 to 20,000. Samples were subsequently gated on live CD45^+^SiglecF^−^Ly6G^−^B220^−^CD3^−^CD64^−^CD11c^+^MHCII^+^ intestinal cDCs or live CD45^+^SiglecF^−^Ly6G^−^B220^−^CD3^+^CD8^−^CD4^+^ and subsampled using a maximum equal distribution across groups. After subsampling, phenograph clustering (*k* = 100) was performed using lineage markers and activation markers in intestinal cDCs and CD4^+^ T cells. Data were further analyzed with EdgeR to determine significant differences in the clusters among different genotypes. OMIQ-exported data for each cell containing the cluster information generated with phenograph were imported into R; heatmaps and volcano plots were generated in R. tSNE analysis was performed using the Flt-SNE algorithm from Kluger laboratory (*92*).

### Quantitative real-time PCR

RNA was extracted from snap-frozen moDCs. The isolation of mRNA was performed using TriPure method. Briefly, 500 μl of TriPure reagent (Sigma-Alrich) and 1 μl of ribonuclease (RNase)–free glycogen (Invitrogen) were added to the pellet of samples. Samples were then vortexed and incubated at RT for 5 min. After this step, 100 μl of RNase-free chlorophorm was added, and samples were inverted vigorously for 30 s. Samples were then centrifuged for 12,000*g* for 15 min at 4°C, and the upper aqueous phase was transferred to a new RNase-free 1.5-ml tube. Then, 250 μl of 2-isopropanol was added to each tube, and after 5 min of incubation, samples were centrifuged for 12,000*g* for 15 min at 4°C. Next, 500 μl of 75% ethanol was added, and tubes were vortexed and centrifuged for 12,000*g* for 15 min at 4°C. As much ethanol as possible was removed and 30 μl of RNase-free MilliQ water was added to the tubes that were incubated for 10 min at 55°C. After this, RNA samples were quantified with NanoDrop (Invitrogen). cDNA was synthesized with reverse transcriptase kit (Promega) using the same amount of RNA for all the samples (settled by the sample with the lowest concentration of RNA). Polymerase chain reaction (PCR) amplification was performed by the SYBR Green method using CFX (Bio-Rad). Glyceraldehyde-3-phosphate dehydrogenase (GAPDH) mRNA levels were used as internal control. Specific primers for detected genes are listed in table S3. Relative expression was determined using the 2^ΔΔCt^ method.

### siRNA electroporation

On day 4 of the DC culture, the cells were harvested and transfected with either 20 nM nontarget control siRNA, 20 nM AMPKα1, 20 nM *ALDH1*, or 20 nM *ALDH2* (all from Dharmacon) or FoxO3 (Invitrogen) siRNA using Neon Transfection System (Invitrogen) with the following setting: 1.600 V, 20-ms width, one pulse. Following electroporation, 1.2 × 10^6^ cells were seeded per well in to a six-well plate containing RPMI media without antibiotics. After 24 hours, culture medium (RPMI) supplemented with 10% FCS, rIL-4 (0.86 ng/ml; R&D Systems) and rGM-CSF (10 ng/ml; Invitrogen) was added. AMPKα1- and FoxO3-silencing efficiency was determined by quantitative PCR, and the transfection efficiency was greater than 90%.

### Western blotting

A minimum of 1 million BM-DCs or moDCs were washed twice with PBS before being lysed in 150 μl of EBSB buffer [8% (w/v) glycerol, 3% (w/v) SDS, and 100 mM tris-HCl (pH 6.8)]. Lysates were immediately boiled for 5 min, and their protein content was determined using a BCA kit. Ten micrograms of protein per lain was separated by SDS–polyacrylamide gel electrophoresis, followed by transfer to a polyvinylidene difluoride membrane. Membranes were blocked for 1 hour at room temperature in TTBS buffer [20 mM tris-HCl (pH 7.6), 137 mM NaCl, and 0.25% (v/v) Tween 20] containing 5% (w/v) fat free milk and incubated overnight with primary antibodies. The primary antibodies used were AMPKα (Cell Signaling Technology, #2532), pAMPK Ser^79^ (Cell Signaling Technology, #2535S), FoxO3 (Cell Signaling Technology, #2497S), pFoxO3 Ser^413^ (Cell Signaling Technology, #8174S), pFoxO3 Ser^253^ (Cell Signaling Technology, #9466), HSP90 (Santa Cruz Biotechnology, #sc7947), and beta-actin (Sigma-Alrich, #A5441). The membranes were then washed in TTBS buffer and incubated with horseradish peroxidase–conjugated secondary antibodies for 2 hours at RT. After washing, blots were developed using enhanced chemiluminescence.

### Enzyme-linked immunosorbent assay

The levels of total TGF-β were measured using a human TGF-β1 duo-set (DY240) with a substrate reagent pack (DY999) according to the manufacturer’s instructions (both R&D Systems Europe, Abingdon, UK). This enzyme-linked immunosorbent assay specifically detects active TGF-β1 and does not cross-react with other subtypes of TGF-β. Total TGF-β1 levels were determined by acid activation (1 M hydrochloric acid, 30 min, RT) of the latent TGF-β1 in the supernatants.

### Digestion of mouse tissues

Lymphoid organs were collected in 500 μl of no additives media (naRPMI = RPMI-1640 supplemented with GlutaMAX (#61870-010 or alternatively 61870036, Gibco, Bleiswijk, Netherlands), in a plate and mechanically disrupted using the back-end of a syringe before addition of 50 μl of a digestion media (dRPMI = naRPMI supplemented with 11× collagenase D (#11088866001, Roche, Woerden, Netherlands; end concentration of 1 mg/ml) and 11× deoxyribonuclease (DNase) I (#D4263, Sigma-Aldrich, Zwijndrecht, Netherlands; end concentration of 2000 U/ml) for 20 min at 37°C and 5% CO_2_. Single-cell suspensions were filtered after digestion with a 100-μm sterile filter (#352360, BD Biosciences, Vianen, Netherlands) before counting in complete RMPI [cRPMI = naRPMI supplemented with 10% heat-inactivated FCS (#S-FBS-EU-015, Serana, Pessin, Germany), 25 nM β-mercaptoethanol (#M6250, Sigma-Aldrich), penicillin (100 U/mL; #16128286, Eureco-pharma, Ridderkerk, Netherlands; purchased inside the LUMC), and streptomycin (100 μg/ml; #S9137, Sigma-Aldrich)]. All mesenteric lymph nodes along the chain from a single mouse were pooled for downstream analyses. Spleens were subjected to red blood cell lysis [in-house; 0.15 M NH_4_Cl, 1 mM KHCO_3_, 0.1 mM EDTA (#15575-038, Thermo, Waltham, MA, USA) in ddH_2_O] for 2 min at room temperature before counting.

### Small intestine and colon lamina propria isolation

Intestinal LP cells were isolated, as previously described (*93*). Briefly, intestine was collected, and fat tissue and peyer’s patches were manually removed. Small intestine was opened longitudinally and feces and mucus were removed using PBS, and tissues were cut in small pieces of ∼2 cm and transferred to a 50-ml tube with HBSS no Ca^2+^ or Mg^2+^ (Gibco) supplemented with 10% FCS and 2 mM EDTA. The pieces of intestine were manually shaken for 30 s, poured into a nitex nylon mesh, and washed again with plain 10 ml of HBSS no Ca^2+^ or Mg^2+^ (Gibco). Tissues were then transferred back to 50-ml tubes, now containing 10 ml of prewarmed HBSS no Ca^2+^ or Mg^2+^ (Gibco) + 2 mM EDTA and placed in an orbital shaker at 200 rpm for 20 min at 37°C. This procedure was repeated three more times, and after the third washing, guts were transferred to a new 50-ml tube containing 1× digestion mix [RPMI GlutaMAX containing 2% FCS, collagenase VIII (1 mg/mL; Sigma-Aldrich) and DNase I (40 U/ml; Sigma-Aldrich)] and incubated for 20 min at 37°C in an orbital shaker at 200 rpm in the case of siLP. Colon tissues were transferred to a new 50-ml tubes containing 1× digestion mix [RPMI GlutaMAX containing 2% FCS, 1 mg/mL of Collagenase IV (Sigma-Aldrich), collagenase D (0.5 mg/ml; Roche), dispase (1 mg/ml; Sigma-Aldrich), and DNase I (40 U/ml; Sigma-Aldrich)] and incubated for 20 min at 37°C in an orbital shaker at 200 rpm After digestion, 35 ml of PBS containing 2% FCS and 2 mM EDTA was added to the samples and placed in a 100-μm cell strainer on top of a new 50-ml tube. Samples were centrifuged at 450*g* for 5 min, resuspended in 10 ml of PBS containing 2% FCS and 2 mM EDTA, placed in a 40-μm cell strainer, and centrifuged at 450*g* for 5 min.

### *H. polygyrus* infection

Mice were infected with *H. polygyrus* with 200 L3 larvae by oral gavage. Tissues were harvested 7 or 21 days postinfection for immunological analysis. Fecal egg count was performed on feces of 21 days infected mice, and it was calculated as egg per gram (epg) of the fecal material. In brief, feces were dissolved in 2 ml of water and left in the fridge overnight. The next day, 2 ml of saturated salt solution was added and mixed thoroughly with Pasteur pipette. Approximately 150 ml of suspension was introduced in a McMaster chamber and viewed under a light microscope (10× objective). The epg was calculated according to the equation: number of eggs counted/volume counted/weight of fecal material × total volume.

### *S. mansoni* acute and chronic infection

Mice were infected with *S. mansoni* (Puerto Rican strain; Naval Medical Research Institute) by 30 min of percutaneous exposure to 35 cercariae on shaved abdomen. Mice were culled 8 (acute infection) or 16 (chronic infection) weeks later. Cercariae were kept at store bought Barleduc water (30 cercariae/ml), which was kept very carefully at 31°C. Female mice were anesthetized by intraperitoneal (i.p.) injection with 300 μl of ketamine (50 mg/kg bodyweight) + dexdomitor (0.5 mg/kg bodyweight), while male mice were anesthetized with ketamine (50 mg/kg bodyweight) + xylazine (10 mg/kg bodyweight). Female mice were assisted in waking up by intraperitoneal injection with 150 μl of 0.4 mg/kg bodyweight. All injections were done using PBS and a 25-gague needle. All anesthetics were purchased at the LUMC pharmacy. Livers were processed same as spleens except that single-cell suspensions were centrifuged twice at 20*g* for 10 min in PBS to remove hepatocytes before red blood cell lysis.

### Cytometric bead array

Cell culture supernatants were analyzed for IFN-γ, IL-4, IL-5, IL-10, and IL-13 secretion using a cytokine bead array (#I558296, #558298, #558302, #558300, and #558349, respectively, and all BD Biosciences) on a flow cytometer as recommended by the manufacturer, but with both the beads and antibodies diluted 1:10 relative to the original recommendation.

### Adenine nucleotides concentration

The intracellular concentration of adenosine mono, di or triphosphate (AMP, ADP, and ATP, respectively) were evaluated as previously described (*94*). Briefly, cells were harvested, transferred to a 1.5-ml tube, and washed in ice-cold PBS. After centrifugation, cells were rinsed with 250 ml of perchloric acid/EDTA [10% (v/v) HClO_4_ and 25 mM EDTA], vortexed, and kept on ice for 30 min. Then, cell extracts were spun down at 8000*g* for 2 min at 4°C, and 200 ml of the supernatant fraction was transferred into a new 1.5-ml tube (on ice), where cells lysate was neutralized by adjusting the pH to 6.5 to 7 with ∼130 ml of KOH/-Mops (2 N KOH/0.3 M Mops). Lysate was centrifuged at 8000*g* for 2 min at 4°C, and 200 ml of the neutralized fraction was transferred to a new 1.5-ml tube and stored in −80°C until measurement of AMP, ADP, and ATP at ultraviolet-based HPLC (DIONEX UltiMate 3000, Thermo Fisher Scientific).

### In silico TF prediction analysis

Prediction of phosphorylated sites in FoxO3 protein by AMPK was performed using GPS 5.0 software (*43*). Briefly, FASTA sequencing data from FoxO3 proteins was downloaded from UniProt website and placed in GPS 5.0 software with a medium threshold selection. The top rank phosphorylated sites targeted by AMPK above the software calculated threshold were selected and pasted in GraphPad for image generation.

### Statistical analysis

Prior sample size determination, randomization and blinding were not performed for in vivo studies. Results are expressed as means ± SEM except stated otherwise. Continuous variables were log-transformed for the analyses when the normality of the distribution was rejected by the Shapiro-Wilk *W* test, and in case of a new normality test failure, the nonparametric alternative was used for the analysis. Differences between groups were analyzed by two-way analysis of variance (ANOVA) (for repeated measurements in case of silenced or compound-treat human DC versus RA-DC) with Sidak’s multiple comparison posttest. If there were only two groups, unpaired (in case of CD11c^WT^ and CD11c^ΔAMPKα1^ mice comparison) or paired (in case of human DC versus RA-DC comparison) Student’s *t* test or Mann-Whitney test (nonparametric analysis) were used. *P* values < 0.05 were considered significant (*P < 0.05, **P < 0.01, and ***P < 0.001), and statistical analyzes were performed using GraphPad Prism v.10.
