# Supplementary material for: Metabolic sensor AMPK licenses intestinal CD103+ DCs to induce Treg responses
Source: Sci Adv. 2026 Sep 16;12(38):eaeb4813. doi: 10.1126/sciadv.aeb4813 (PMC13580595; doi:10.1126/sciadv.aeb4813)
Supplement: Supplementary file 1 [file sciadv.aeb4813_sm.pdf]

Science Advances
AAAS

Supplementary Materials for

Metabolic sensor AMPK licenses intestinal CD103$^+$ DCs to induce T_{reg} responses

Thiago A. Patente *et al.*

Corresponding author: Bart Everts, b.everts@lumc.nl

Sci. Adv. **12**, eaeb4813 (2026)
DOI: 10.1126/sciadv.aeb4813

The PDF file includes:

Figs. S1 to S6
Tables S1 to S3
Legend for data file S1

Other Supplementary Material for this manuscript includes the following:

Data file S1

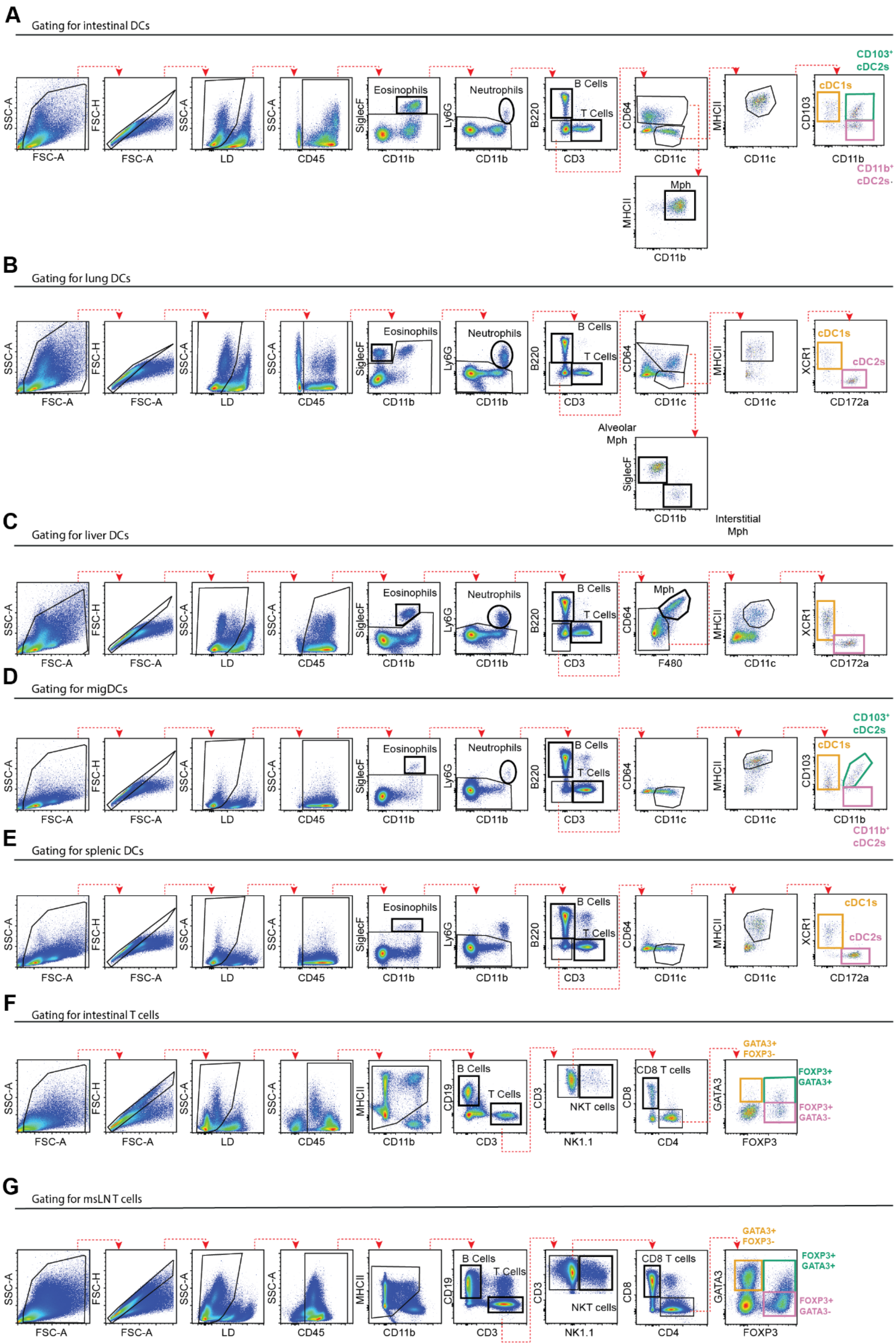

A
Gating for intestinal DCs
SSC-A
FSC-A
FSC-H
LD
CD45
SiglecF
Eosinophils
CD11b
Ly6G
Neutrophils
B220
B Cells
T Cells
CD3
CD64
CD11c
MHCII
Mph
CD103
cDC1s
CD103+ cDC2s
CD11b+ cDC2s
B
Gating for lung DCs
Alveolar Mph
Interstitial Mph
XCR1
cDC2s
CD172a
C
Gating for liver DCs
F480
D
Gating for migDCs
E
Gating for splenic DCs
F
Gating for intestinal T cells
CD19
NK1.1
NKT cells
CD8
CD8 T cells
CD4
GATA3
FOXP3
GATA3+ FOXP3-
FOXP3+ GATA3+
FOXP3+ GATA3-
G
Gating for msLN T cells

Fig. S1: Gating strategy used in different tissues to analyze *in vivo c*DCs subsets.

Representative staining and gating strategies for cDC1s, CD103$^+$ cDC2s, CD11b$^+$ cDC2s and macrophages in small intestine lamina propria (siLP) **(A)**, cDC1s, cDC2s, alveolar and interstitial macrophages in lungs **(B)**, cDC1s, cDC2s and Kupfer cells in liver **(C)**, migratory cDC1s, CD103$^+$ cDC2s, CD11b$^+$ cDC2s in mesenteric lymph nodes **(D)** and cDC1s and cDC2s in spleen **(E)**. Representative staining and gating strategies for T cell populations in small intestine lamina propria (siLP) **(F)** and mesenteric Ln **(G)**.

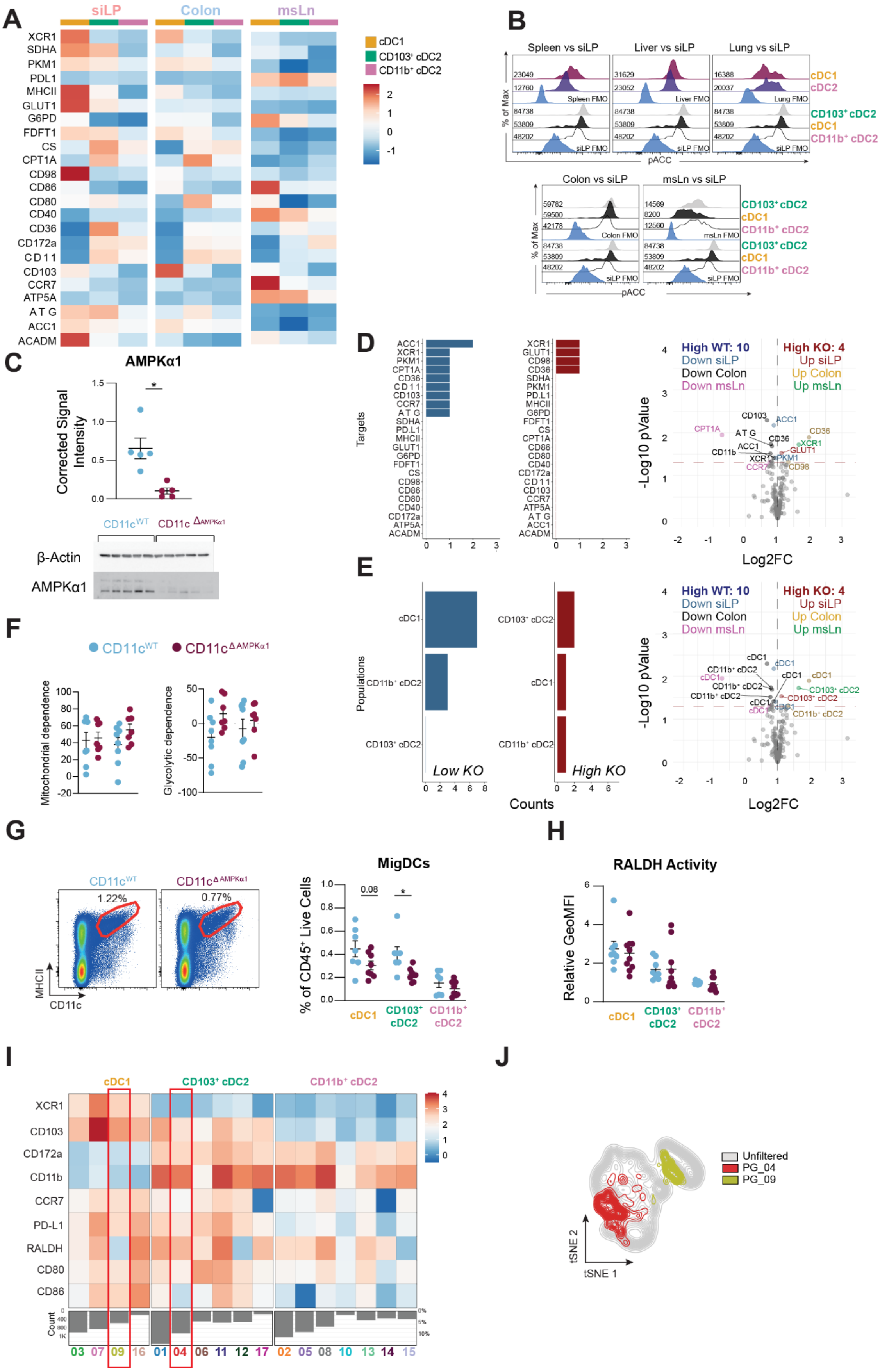
A
siLP
Colon
msLn
XCR1
SDHA
PKM1
PDL1
MHCII
GLUT1
G6PD
FDFT1
CS
CPT1A
CD98
CD86
CD80
CD40
CD36
CD172a
CD11
CD103
CCR7
ATP5A
AT G
ACC1
ACADM
cDC1
CD103⁺ cDC2
CD11b⁺ cDC2
2
1
0
-1
B
Spleen vs siLP
Liver vs siLP
Lung vs siLP
% of Max
Spleen FMO
Liver FMO
Lung FMO
siLP FMO
pACC
cDC1
cDC2
CD103⁺ cDC2
CD11b⁺ cDC2
Colon vs siLP
msLn vs siLP
Colon FMO
msLn FMO
C
AMPKα1
Corrected Signal Intensity
1.5
1.0
0.5
0.0
*
CD11c^WT
CD11c ^ΔAMPKα1
β-Actin
AMPKα1
D
Targets
High WT: 10
High KO: 4
Down siLP
Up siLP
Down Colon
Up Colon
Down msLn
Up msLn
-Log10 pValue
Log2FC
E
Populations
Low KO
High KO
Counts
F
CD11c^WT
CD11c^ΔAMPKα1
Mitochondrial dependence
Glycolytic dependence
G
CD11c^WT
CD11c^ΔAMPKα1
1.22%
0.77%
MHCII
CD11c
MigDCs
% of CD45⁺ Live Cells
0.08
*
cDC1
CD103⁺ cDC2
CD11b⁺ cDC2
H
RALDH Activity
Relative GeoMFI
I
cDC1
CD103⁺ cDC2
CD11b⁺ cDC2
XCR1
CD103
CD172a
CD11b
CCR7
PD-L1
RALDH
CD80
CD86
Count
03 07 09 16 01 04 06 11 12 17 02 05 08 10 13 14 15
J
Unfiltered
PG_04
PG_09
tSNE 2
tSNE 1

Fig. S2: Mesenteric lymph node cDC1s and $CD103^+$ cDC2s are reduced in $CD11c^{\Delta AMPK\alpha 1}$ mice.

(A) Heatmap of activation and metabolic markers expressed by intestinal cDC subsets. **(B)** Representative histograms for phosphorylation of Ser79 in acetyl-coA carboxylase (pACC) in cDC subsets across different tissues. Data from Figure 1E. (**C**) Immunoblot for AMPKα on lysates from total CD11c+ splenic cDCs. **(D)** Number of decreased (blue – left) and increased (red – middle) markers expressed by each intestinal cDC subset, and a volcano plot displaying significantly increased and decreased markers (right) from $CD11c^{WT}$ versus $CD11c^{\Delta AMPK\alpha 1}$ mice. **(E)** Number of decreased (blue – left) and increased (red – middle) markers expressed by each intestinal cDC subset, and a volcano plot displaying significantly increased and decreased markers (right). (**F**) SCENITH analysis on cDC subsets from SiLP and mesenteric lymph nodes (msLns) from of $CD11c^{WT}$ and $CD11c^{\Delta AMPK\alpha 1}$ mice. **(G)** Representative dot plot for the gating of migratory DCs (migDCs - top) and frequency of $CD45^+$ live cells of $CD103^+$ DCs, $CD103^+CD11b^+$ DCs, $CD11b^+$ DCs (bottom) in msLNs of $CD11c^{WT}$ and $CD11c^{\Delta AMPK\alpha 1}$ mice. **(H)** Relative geoMFI (R-MFI) of RALDH activity in migDCs subsets in the msLNs of $CD11c^{WT}$ and $CD11c^{\Delta AMPK\alpha 1}$ mice. **(I)** Heatmap displaying activity of RALDH and expression of activation and lineage markers from clusters identified in Figure 1K. **(J)** Contour plot of PG_04 (red) and PG_09 (ochre) displaying tSNE1 vs tSNE2. Data are representative of 1 out of 2 independent experiments **(A,B,,E,I,J)**; or a pool of 2 (**F**) or 3 **(G,H)** independent experiments with 3 to 4 mice per experiment**.** Student's T-test followed by p-value false discovery rate (FDR) **(D,E)**; or Student's t test **(G,H)** was used to assess statistically significant differences. Mean ± SEM are indicated in the graphs;*$p < 0.05$, **$p < 0.01$, ***$p < 0.001$, ****$p<0.0001$.

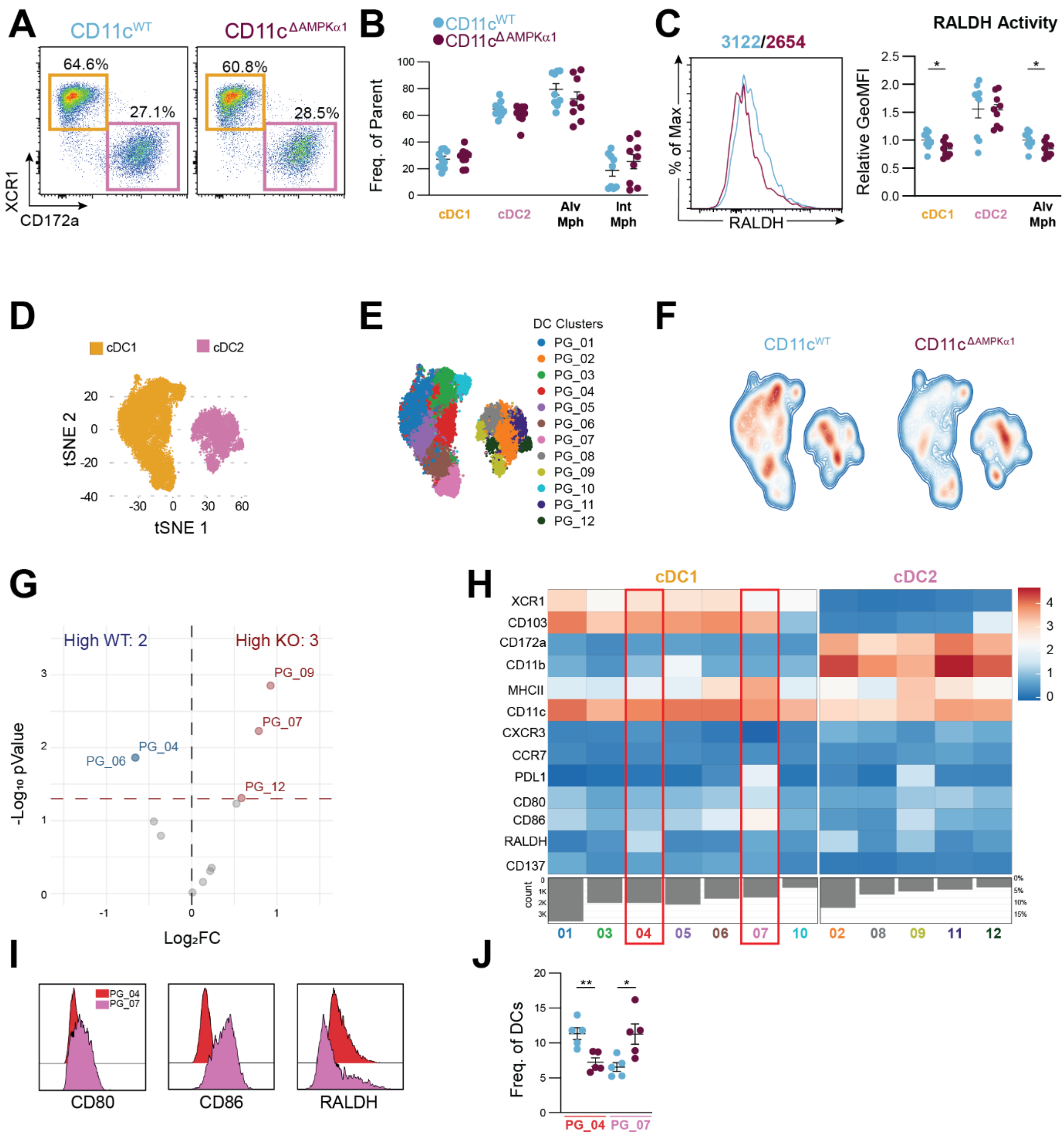
A
CD11c^WT
CD11c^ΔAMPKα1
64.6%
27.1%
60.8%
28.5%
XCR1
CD172a
B
CD11c^WT
CD11c^ΔAMPKα1
Freq. of Parent
cDC1
cDC2
Alv Mph
Int Mph
C
3122/2654
% of Max
RALDH
RALDH Activity
Relative GeoMFI
cDC1
cDC2
Alv Mph
D
cDC1
cDC2
tSNE 2
tSNE 1
E
DC Clusters
PG_01
PG_02
PG_03
PG_04
PG_05
PG_06
PG_07
PG_08
PG_09
PG_10
PG_11
PG_12
F
CD11c^WT
CD11c^ΔAMPKα1
G
High WT: 2
High KO: 3
PG_09
PG_07
PG_04
PG_06
PG_12
-Log₁₀ pValue
Log₂FC
H
cDC1
cDC2
XCR1
CD103
CD172a
CD11b
MHCII
CD11c
CXCR3
CCR7
PDL1
CD80
CD86
RALDH
CD137
count
01 03 04 05 06 07 10 02 08 09 11 12
I
PG_04
PG_07
CD80
CD86
RALDH
J
Freq. of DCs
PG_04
PG_07

Fig. S3: AMPK signaling is important for RALDH activity in lung cDC1s.

(A) Representative dot plot for cDC1 and cDC2 in the lungs of $CD11c^{WT}$ and $CD11c^{\Delta AMPK\alpha 1}$ mice. **(B)** Frequencies of DCs and macrophages subsets and **(C)** Representative histogram (left) and relative geometric MFI (R-MFI) of RALDH activity in the lungs of $CD11c^{WT}$ and $CD11c^{\Delta AMPK\alpha 1}$ mice. **(D)** Unbiased tSNE analysis of lung DC populations in which cDC1s and cDC2s are indicated. **(E)** Phenograph clustering performed on lung DCs using RALDH, activation and lineage markers. **(F)** Contour plots overlaid on tSNE analysis as shown in **(D)** displaying distribution of cells for $CD11c^{WT}$ and $CD11c^{\Delta AMPK\alpha 1}$ mice. **(G)** Volcano plot displaying the clusters as displayed in **(E)** with significant differences in frequency between $CD11c^{WT}$ and $CD11c^{\Delta AMPK\alpha 1}$ mice. **(H)** Heatmap displaying expression of RALDH, activation and lineage markers from clusters identified in **(E)**. **(I)** Histograms displaying the activity of RALDH, or expression levels CD86 and CD80 in PG_04 (red) and PG_07 (pink). **(J)** Frequency of PG_04 (red) and PG_07 (pink) in $CD11c^{WT}$ and $CD11c^{\Delta AMPK\alpha 1}$ mice. Data are a pool of 2 experiments with 7-9 mice **(A-C)**, 1 experiment using 5 mice per group **(D-J).** Student's T-test **(B,C,J)**; or EDGE-R with p-value false discovery rate **(G)** was used to assess statistically significant differences. Mean ± SEM are indicated in the graphs;$^*p < 0.05$, $^{**}p < 0.01$, $^{***}p < 0.001$, $^{****}p<0.0001$.

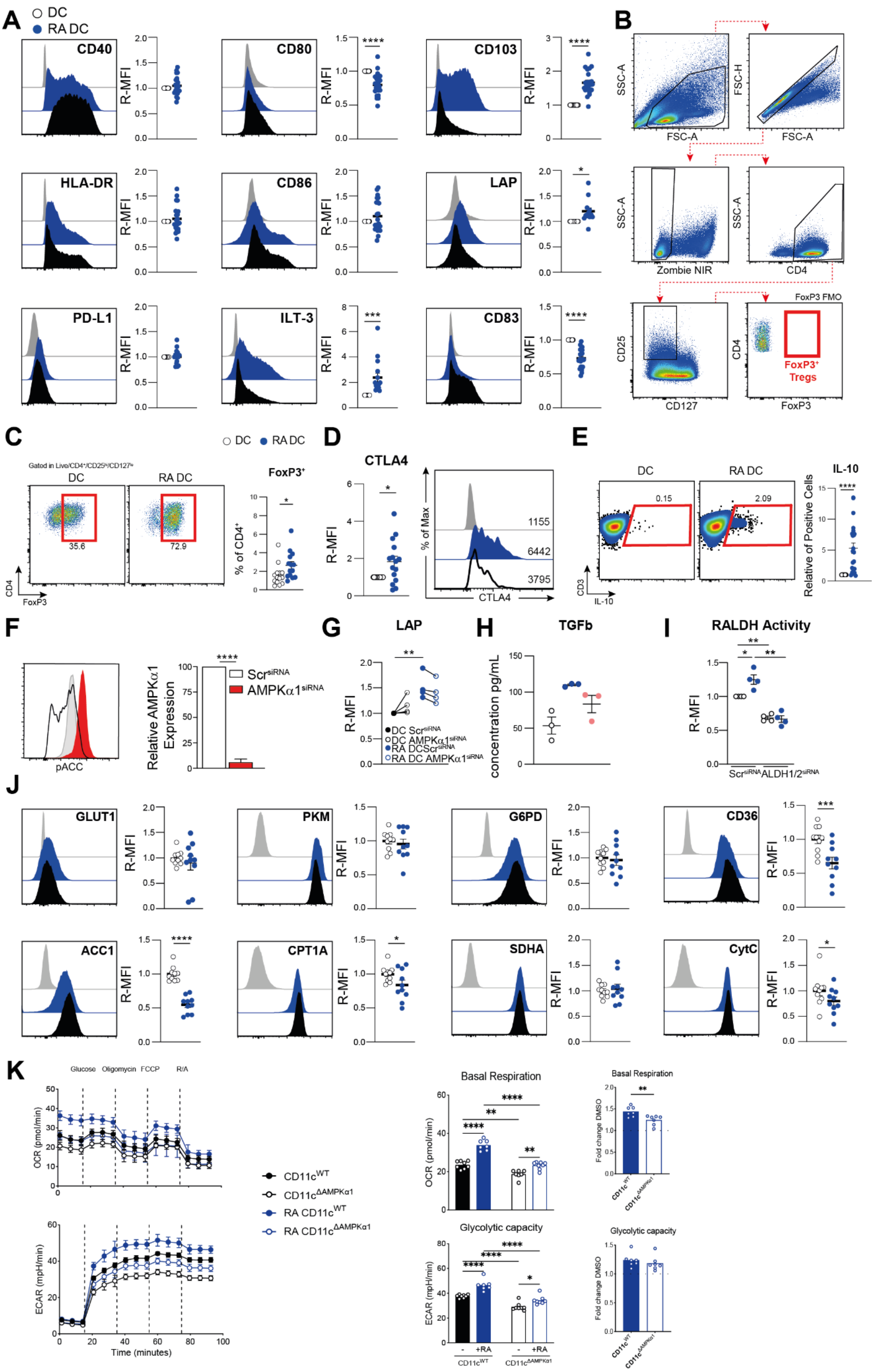

A
DC
RA DC
CD40
CD80
CD103
HLA-DR
CD86
LAP
PD-L1
ILT-3
CD83
R-MFI
B
SSC-A
FSC-A
FSC-H
Zombie NIR
CD4
CD25
CD127
FoxP3 FMO
FoxP3
FoxP3+ Tregs
C
Gated in Live/CD4+/CD25hi/CD127lo
FoxP3+
% of CD4+
D
CTLA4
% of Max
1155
6442
3795
E
IL-10
CD3
Relative of Positive Cells
F
pACC
Relative AMPKα1 Expression
ScrsiRNA
AMPKα1siRNA
G
LAP
DC ScrsiRNA
DC AMPKα1siRNA
RA DCScrsiRNA
RA DC AMPKα1siRNA
H
TGFb
concentration pg/mL
I
RALDH Activity
ALDH1/2siRNA
J
GLUT1
PKM
G6PD
CD36
ACC1
CPT1A
SDHA
CytC
K
Glucose
Oligomycin
FCCP
R/A
OCR (pmol/min)
ECAR (mpH/min)
Time (minutes)
CD11cWT
CD11cΔAMPKα1
RA CD11cWT
RA CD11cΔAMPKα1
Basal Respiration
Glycolytic capacity
Fold change DMSO

Fig. S4: Metabolic and phenotype characterization of tolerogenic human monocyte-derived DCs.

(A,H,I) Monocytes were isolated from PBMC with $CD14^+$ magnetic beads and differentiated in moDCs in the presence of GM-CSF + IL-4. On day 5 cells were treated with either RA or DMSO. On the 6th day, 100 ng/mL of LPS were added and 24 hours later cells were harvested for functional assays. **(A)** Representative histograms staining (left) and quantification levels (right) of the relative geometric mean fluorescence (R-MFI) of CD40, CD80, CD103, HLA-DR, CD86, LAP, PD-L1, ILT3, and CD83 normalized by DC set as 1 (data used to generate heatmap in Figure 4A). **(B)** Gating strategy to gate human $FoxP3^+$ Tregs. (**C-E**) moDCs were co-cultured with allogeneic naïve T cells and intracellular (**C**) FoxP3 staining, (**D**) level of CTLA4 and (**E**) levels of IL-10 were measured by flow cytometry after7 days of co-culture and 5 additional days of T cells expansion. (**C**) Frequencies in the left flow plots are % of parent gate ($CD127^-CD25^+CD4^+$ T cells). **(F)** pACC staining and mRNA expression levels of AMPKα1 in moDCs silenced with scramble (white) or siRNA targeting AMPKα1 (red). **(G)** moDCs were silenced for AMPKα1 or non-targeting scramble RNA, and 24 hrs later cells were treated with RA. Next, moDCs were treated with LPS and 24hrs later they were harvested for evaluation of LAP expression (as a TGF-β readout). (**H**) TGF-β was quantified by ELISA in supernatants of moDCs stimulated for 24h with LPS with (blue) or without RA (white) in the presence or absence of AMPK inhibitor BAY-3827 (pink). Data are normalized relative to untreated control conditions **(I)** Monocytes were isolated from PBMC with $CD14^+$ magnetic beads and differentiated in moDCs in the presence of GM-CSF + IL-4. On day 4 cells were transfected with small interfering RNA (siRNA) against ALDH1 and ALDH2 and 24 hours later cells were treated with either RA or left untreated. On the 6th day, 100 ng/mL of LPS were added and 24 hours later cells were harvested. Quantification of the R-MFI of RALDH activity from DC and RA-DC silence with either scramble siRNA or ALDH1/2 siRNA **(J)** Representative histogram staining (left) and quantification levels (right) GLUT1, PKM, G6PD, CD36, ACC1, CPT1A, CytC and SDHA. (**K**) $CD11b^+$ cDC2s purified from Flt3L BM cultures from $CD11c^{WT}$ and $CD11c^{\Delta AMPK\alpha 1}$ mice were stimulated with RA and 24h later analysed by SEAHORSE metabolic flux analysis to assess rates of glycolysis (ECAR) and oxphos (OCR). Data are pooled from 15-22 donors **(A-E)**, 3 donors (**H**) 4 donors **(F,G,I)**, 10 donors **(J)** or 7 mice **(K)** . Statistics are paired Student's T-test **(A,C-E,I)** or Two-Way Anova for repeated measurements with Sidaks post-test. Data shown as mean ± SEM; *p < 0.05, **p < 0.01, ***p < 0.001, ****p<0.0001.

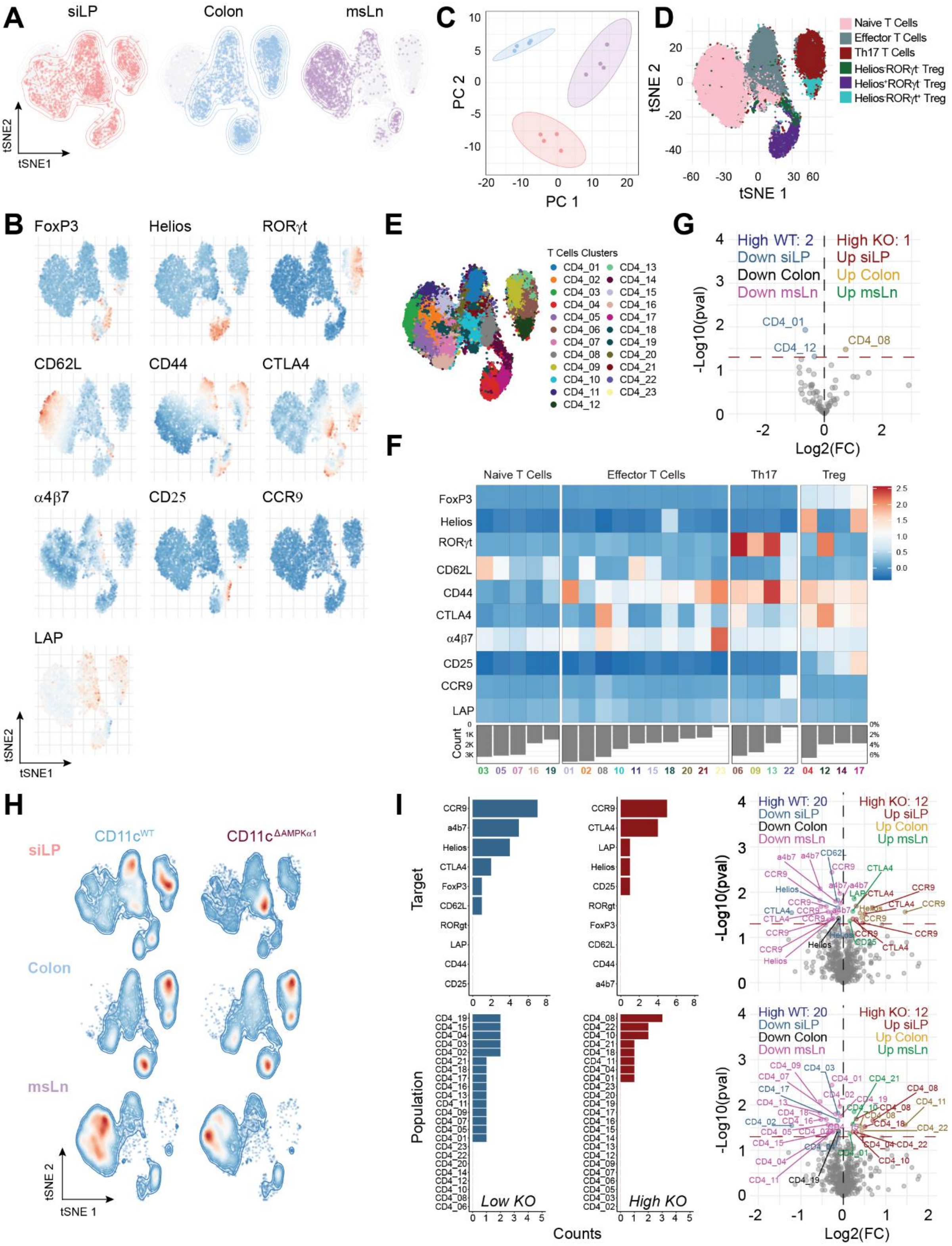
A
siLP
Colon
msLn
tSNE1
tSNE2
B
FoxP3
Helios
RORγt
CD62L
CD44
CTLA4
α4β7
CD25
CCR9
LAP
C
PC 1
PC 2
D
Naive T Cells
Effector T Cells
Th17 T Cells
Helios⁻RORγt⁻ Treg
Helios⁺RORγt⁻ Treg
Helios⁻RORγt⁺ Treg
tSNE 1
tSNE 2
E
T Cells Clusters
F
Naive T Cells
Effector T Cells
Th17
Treg
Count
G
High WT: 2
High KO: 1
Down siLP
Up siLP
Down Colon
Up Colon
Down msLn
Up msLn
CD4_01
CD4_12
CD4_08
-Log10(pval)
Log2(FC)
H
siLP
Colon
msLn
CD11c^WT
CD11c^ΔAMPKα1
tSNE 1
tSNE 2
I
Target
Population
Low KO
High KO
Counts
High WT: 20
High KO: 12
Down siLP
Up siLP
Down Colon
Up Colon
Down msLn
Up msLn
-Log10(pval)
Log2(FC)

Figure S5: AMPK signaling in CD11c-expressing cells is dispensable for intestinal Treg homeostasis at steady-state

(A) UMAP of $CD4^+$ T cells from siLP, colon and msLn. **(B)** UMAP displaying expression of different lineage and activation markers. **(C)** Principal component analysis (PCA) of intestinal $CD4^+$ T cells determined by the expression pattern of lineage and activation markers. **(D)** Unbiased tSNE analysis of intestinal $CD4^+$ T cells populations in which naïve, antigen-experienced, Th17, $Helios^-ROR\gamma t^-$, $Helios^+ROR\gamma t^-$ and $Helios^-ROR\gamma t^+$ Tregs are indicated. **(E)** Contour plots overlaid on tSNEs as shown in (D) displaying distribution of T cells in siLP, colon and msLn for $CD11c^{WT}$ and $CD11c^{\Delta AMPK\alpha 1}$ mice. **(F)** Phenograph clustering performed on intestinal $CD4^+$ T cells lineage and activation markers. **(G)** Volcano plot displaying the clusters as displayed in **(F)** with significant differences in frequency between $CD11c^{WT}$ and $CD11c^{\Delta AMPK\alpha 1}$ mice. **(H)** Heatmap displaying the expression of different markers within each cluster identified in (F). **(I)** Contour plot of CD4_08 displaying CTLA4 and CD44 (left) and histogram of CTLA4 (right) expression for $CD11c^{WT}$ and $CD11c^{\Delta AMPK\alpha 1}$ mice. Data are representative of 1 out of 2 independent experiments with 3-4 mice per genotype and per experiment. EDGE-R was used to calculate adjusted p-value for differences in clustering frequencies amongst genotypes **(G)**.

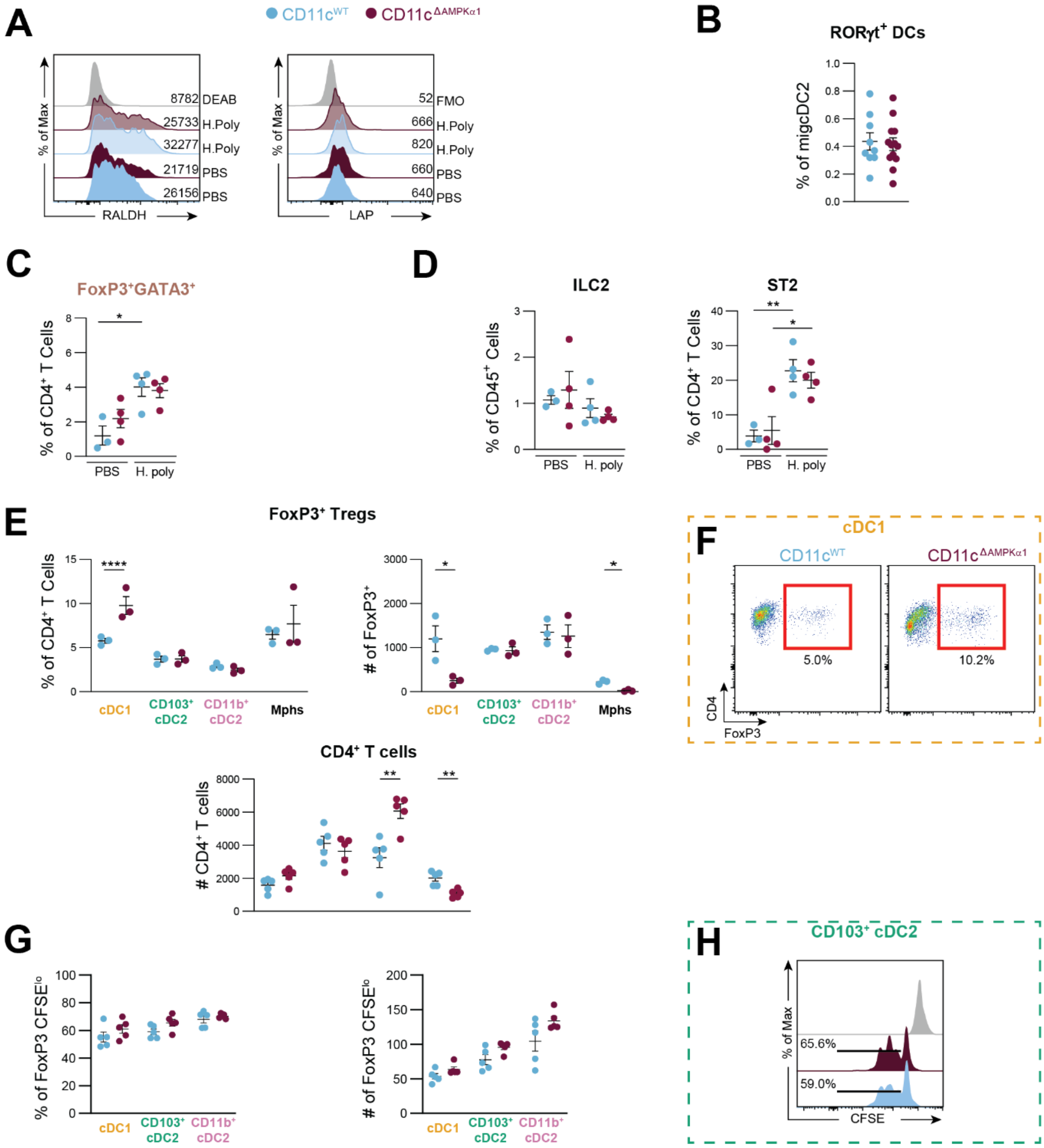
A
CD11c^WT CD11c^ΔAMPKα1
% of Max
8782 DEAB
25733 H.Poly
32277 H.Poly
21719 PBS
26156 PBS
RALDH
52 FMO
666 H.Poly
820 H.Poly
660 PBS
640 PBS
LAP
B
RORγt+ DCs
% of migcDC2
C
FoxP3+GATA3+
% of CD4+ T Cells
PBS
H. poly
D
ILC2
% of CD45+ Cells
ST2
% of CD4+ T Cells
E
FoxP3+ Tregs
% of CD4+ T Cells
# of FoxP3+
cDC1
CD103+ cDC2
CD11b+ cDC2
Mphs
CD4+ T cells
CD4+ T cells
F
cDC1
CD11c^WT
CD11c^ΔAMPKα1
5.0%
10.2%
CD4
FoxP3
G
% of FoxP3 CFSE^lo
# of FoxP3 CFSE^lo
H
CD103+ cDC2
% of Max
65.6%
59.0%
CFSE

Fig. S6: ex vivo Treg priming capacity of intestinal cDCs subsets.

(A) Representative histograms of RALDH activity (left) and LAP expression (right) in the siLP and msLn, respectively, of $CD103^+$ cDC2s from naïve and infected $CD11c^{WT}$ and $CD11c^{\Delta AMPK\alpha 1}$ mice. **(B)** Frequency of $ROR\gamma t^+$ DCs 7 days post infection with *H. polygyrus* in the msLn of $CD11c^{WT}$ and $CD11c^{\Delta AMPK\alpha 1}$ mice. **(C)** Representative dot plots (upper) and quantification of frequency of $FoxP3^+$ Treg subsets from SiLP of $CD11c^{WT}$ and $CD11c^{\Delta AMPK\alpha 1}$ mice 7 days post infection with *H. polygyrus.* (**D**) Frequency of $ST2^+$ Th2 cells and $CD3^-IL7R^+GATA3^+$ ILC2s 21 days post infection with *H. polygyrus* in the msLn of $CD11c^{WT}$ and $CD11c^{\Delta AMPK\alpha 1}$ mice. **(E-H)** $CD64^+CD11c^+$ Mphs and intestinal DCs subsets were sorted by flow cytometry, pulsed with OVA in the presence of LPS and CpG-B for 2hrs, and co-cultured in a 1:5 ratio with naïve OT-II T cells in the presence of TGFb for evaluation of Treg priming. **(E)** Frequency of CD4 (left) and cell numbers (right) of $FoxP3^+$ Tregs primed by sorted siLP derived cDC1s, $CD103^+$ cDC2s, $CD11b^+$ cDC2 or Mphs. **(F)** Representative dot plots of $FoxP3^+$ Tregs primed by cDC1s. **(G)** Frequency of $FoxP3^+$ (left) and cell numbers (right) of $FoxP3^+CFSE^{lo}$ Tregs primed by sorted siLP-derived cDC1s, $CD103^+$ cDC2s or $CD11b^+$ cDC2. **(H)** Representative histograms of $FoxP3^+$ Tregs proliferation primed by $CD103^+$ cDC2s the siLP. Data are a pool of 2 independent experiments with 4-8 mice per group **(A--D)**; or representative of 1 out 2 independent experiments with 3 mice per group in each experiment **(E,F)**, from 1 experiment using 4 mice per group **(G,H)**. Student's T-test **(A,E,G)**. Mean ± SEM are indicated in the graph.; $^*p < 0.05$, $^{**}p < 0.01$, $^{***}p < 0.001$, $^{****}p<0.0001$.

Table S1: Antibody list

Antibodies	Source	Identifier
Anti-human CD103 (Clone Ber-ACT8)	BioLegend	Cat# 350211
Anti-human CD127 (Clone eBioRDR5)	Invitrogen/eBioscience	Cat# 47-1278-42
Anti-human CD14 (Clone MΦP9)	BD Biosciences	Cat# 345786
Anti-human CD152 (CTLA-4) (Clone BNI3)	BD Biosciences	Cat# 555853
Anti-human CD1a (Clone HI149)	BD Biosciences	Cat# 555806
Anti-human CD1a (Clone HI149)	BioLegend	Cat# 300127
Anti-human CD25 (Clone M-A251)	BD Biosciences	Cat# 562442
Anti-human CD3 (Clone UCHT1)	Invitrogen/eBioscience	Cat# 47-0038-42
Anti-human CD4 (Clone SK3)	Invitrogen/eBioscience	Cat# 46-0047-42
Anti-human CD40 (Clone 5C3)	BD Biosciences	Cat# 555591
Anti-human CD40 (Clone 5C3)	BioLegend	Cat# 334338
Anti-human CD80 (Clone L307,4)	BD Biosciences	Cat# 560442
Anti-human CD83 (Clone HB15e)	Invitrogen/eBioscience	Cat# 12-0839-42
Anti-human CD85k (ILT3) (Clone ZM4.1)	BioLegend	Cat# 333015
Anti-human CD86 (Clone 2331 (FUN-1))	BD Biosciences	Cat# 561128
Anti-human CD86 (Clone 2331 (FUN-1))	BD Biosciences	Cat# 555657
Anti-human FoxP3 (Clone PCH101)	Invitrogen/eBioscience	Cat# 17-4776-41
Anti-human HLA-DR (Clone LN3)	Invitrogen/eBioscience	Cat# 47-9956-42
Anti-human IL-10 (Clone JES3-19F1)	BD Biosciences	Cat# 554707
Anti-human IL-13 (Clone JES10-5A2)	BioLegend	Cat# 501903
Anti-human IL-17a (Clone BL168)	BioLegend	Cat# 512304
Anti-human IL-4 (Clone 8D4-8)	eBioscience	Cat# 25-7049-82
Anti-human INF-g (Clone B27)	BD Biosciences	Cat# 560371
Anti-human LAP (Clone TW4-2F8)	BioLegend	Cat# 349611
anti-mouse a4b7 (Clone DATK32)	BD	Cat# 740892
Anti-mouse B220 (Clone RA3-6B2)	BioLegend	Cat# 103248
Anti-mouse B220 (Clone RA3-6B2)	BD Bioscience	Cat# 553091
anti-mouse CCR6 (Clone 140706)	BD	Cat# 740646
anti-mouse CCR9 (Clone eBioCW-1.2)	eBioscience	Cat# 17-1991-80
Anti-mouse CD103 (Clone 2E7)	BD Biosciences	Cat# 750718
Anti-mouse CD103 (Clone 2E7)	Invitrogen/eBioscience	Cat# 13-1031-85
Anti-mouse CD11b (Clone M1/70)	Invitrogen/eBioscience	Cat# 25-0112-82
Anti mouse CD11b (Clone M1/70)	BD Bioscience	Cat# 569711
Anti-mouse CD11c (Clone N418)	BioLegend	Cat# 117330
anti-mouse CD127 (Clone A7R34)	BD	Cat# 569065
Anti-mouse CD152 (CTLA-4) (Clone UC10-4B9)	BD Bioscience	Cat# 12-1522-82
Anti-mouse CD152 (CTLA-4) (Clone UC10-4B9)	BioLegend	Cat# 106312
Anti-mouse CD172a (Clone P84)	BioLegend	Cat# 144011
Anti-mouse CD197 (CCR7) (Clone 4B12)	BioLegend	Cat# 120125
Anti-mouse CD25 (Clone PC61.5)	BD Bioscience	Cat# 551071
Anti-mouse CD25 (Clone PC61.5)	BioLegend	Cat# 102010
Anti-mouse CD274 (PD-L1) (Clone 10F.9G2)	BioLegend	Cat# 124319
Anti-mouse CD3 (Clone 17A2)	Invitrogen/eBioscience	Cat# 11-0032-82
Anti-mouse CD3 (Clone 17A2)	Invitrogen/eBioscience	Cat# 48-0032-82

Anti-mouse CD3 (Clone 17A2)	BioLegend	Cat# 100237
Anti-mouse CD357 (GITR) (Clone YGITR 765)	BioLegend	Cat# 120222
Anti-mouse CD4 (Clone GK1.5)	Invitrogen/eBioscience	Cat# 25-0041-81
Anti-mouse CD4 (Clone GK1.5)	Invitrogen/eBioscience	Cat# 46-0041-82
Anti-mouse CD4 (Clone GK1.5)	BD Biosciences	Cat# 563232
anti-mouse CD40 (Clone 3/23)	BD	Cat# 756113
Anti-mouse CD40 (Clone HM40-3)	Invitrogen/eBioscience	Cat# 11-0402-82
Anti-mouse CD44 (Clone IM7)	Invitrogen/eBioscience	Cat# 48-0441-82
Anti-mouse CD44 (Clone IM7)	Invitrogen/eBioscience	Cat# 25-0441-81
Anti-mouse CD45 (Clone 30-F11)	BD Bioscience	Cat# 748370
Anti-mouse CD45 (Clone 30-F11)	BD Bioscience	Cat# 564225
Anti-mouse CD45 (Clone 30-F11)	BioLegend	Cat# 103173
Anti-mouse CD62L (Clone MEL-14)	Invitrogen/eBioscience	Cat# 47-0621-82
Anti-mouse CD64 (Clone X54-5/7.1)	BioLegend	Cat# 139304
Anti-mouse CD64 (Clone X54-5/7.1)	BioLegend	Cat# 139320
Anti-mouse CD64 (Clone X54-5/7.1)	BioLegend	Cat # 139311
Anti-mouse CD80 (Clone 16-10A1)	BioLegend	Cat# 104714
anti-mouse CD80 (Clone 16-10A1)	Biolegend	Cat# 104734
anti-mouse CD80 (Clone 16-10A1)	Biolegend	Cat# 104762
Anti-mouse CD86 (Clone GL-1)	BD Bioscience	Cat# 560581
anti-mouse CD86 (Clone PO3)	BD	Cat# 750437
Anti-mouse CD8a (Clone 53-6.7)	Tonbo Biosciences	Cat# 20-0081-U025
Anti-mouse CD8a (Clone 53-6.7)	BioLegend	Cat# 100714
Anti-mouse CD8a (Clone 53-6.7)	BioLegend	Cat# 100747
anti-mouse CD90 (Clone HIS51)	BD	Cat# 751156
anti-mouse CTLA4 (Clone UC10-4B9)	Biolegend	Cat# 106335
Anti-mouse CX3CR1 (Clone SA011F11)	BioLegend	Cat# 149025
anti-mouse CXCR6 (Clone SA051D1)	Biolegend	Cat# 151111
anti-mouse EpCAM (Clone 68.8)	Biolegend	Cat# 118227
anti-mouse ESAM (Clone 1G8)	BD	Cat# 752446
Anti-mouse F4/80 (Clone BM8)	Invitrogen/eBioscience	Cat# 47-4801-82
Anti-mouse FoxP3 (Clone FJK-16s)	Invitrogen/eBioscience	Cat# 12-5773-82
Anti-mouse FoxP3 (Clone FJK-16s)	Invitrogen/eBioscience	Cat# 17-577382
anti-mouse GATA3 (Clone TWAJ)	Invitrogen/eBioscience	Cat# 46-9966-42
Anti-mouse Helios (Clone 22F6)	BioLegend	Cat# 137213
anti-mouse ICOS (Clone 15F9)	Biolegend	Cat# 107716
anti-mouse ICOS (Clone C398.4A)	Biolegend	Cat# 313520
Anti-mouse IFNγ (Clone XMG1.2)	Invitrogen/eBioscience	Cat# 25-7311-82
Anti-mouse IFNγ (Clone XMG1.2)	Invitrogen/eBioscience	Cat# 11-7311-41
Anti-mouse IL-10 (Clone JES5-16E3)	Invitrogen/eBioscience	Cat# 12-7101-82
Anti-mouse IL-12p40/p70 (Clone C15.6)	Invitrogen/eBioscience	Cat# 554480
anti-mouse IL-13 (Clone ebio13A)	eBioscience	Cat# 25-7133-82
Anti-mouse IL-17A (Clone eBio17B7)	Invitrogen/eBioscience	Cat# 45-7177-80
Anti-mouse IL-4 (Clone 11B11)	Invitrogen/eBioscience	Cat# 17-7041-82
anti-mouse IL-5 (Clone TRFK5)	Biolegend	Cat# 504304
anti-mouse Ki67 (Clone 16A8)	Biolegend	Cat# 652413
Anti-mouse LAP (Clone Tw7-16B4)	Invitrogen/eBioscience	Cat# 46-9821-82

Anti-mouse Ly6C (Clone HK1.4)	BioLegend	Cat# 128025
Anti-mouse Ly6C (Clone HK1.4)	Invitrogen/eBioscience	Cat# 45-5932-80
Anti-mouse Ly6G (Clone 1A8)	BioLegend	Cat# 108467
Anti-mouse MERTK (Clone 2B10C42)	BioLegend	Cat# 151507
Anti-mouse MHC II (Clone 2G9)	BD Bioscience	Cat# 743876
Anti-mouse MHCII (Clone M5/114.15.2)	Invitrogen/eBioscience	Cat# 56-5321-80
Anti-mouse MHCII (Clone M5/114.15.2)	Invitrogen/eBioscience	Cat# 47-5321-82
anti-mouse NCAM-1 (Clone 809220)	BD	Cat# 748095
anti-mouse Nk1.1 (Clone S17016D)	Biolegend	Cat# 156528
anti-mouse PDL2 (Clone 122)	Invitrogen	Cat# 46-9972-82
Anti-mouse RORgt (Clone Q31-378)	BD Bioscience	Cat# 562607
Anti-mouse Siglec-F (Clone E50-2440)	BD Biosciences	Cat# 740388
Anti-mouse Siglec-H (Clone 440c)	BD Biosciences	Cat# 747675
Anti-mouse Siglec-H (Clone 511)	BioLegend	Cat# 129611
anti-mouse ST2 (Clone 429-93)	BD	Cat# 753456
anti-mouse TIGIT (Clone 1G9)	Biolegend	Cat# 142110
Anti-mouse TNF-a (Clone MP6-XT22)	BioLegend	Cat# 506324
Anti-mouse XCR1 (Clone ZET)	BioLegend	Cat# 148220
Goat anti-rabbit (Clone NA)	Invitrogen	Cat# A27040
Streptavidin (Clone NA)	BioLegend	Cat# 405214
Streptavidin (Clone NA)	BioLegend	Cat# 405249

Table S2: Metabolic antibody list

Target	Fluor	Source	Identifier. #
Metabolic Targets			
Phospho-Acetyl-CoA Carboxylase (Ser79)	-	Cell Signaling Technology	#11818
Phospho-S6 Ribosomal Protein (Ser240/244)	PE	Cell Signaling Technology	#14236
GLUT1<br>*(Lightning link)*	BSA/Azide free<br>DL405	Abcam	ab252403<br>ab201798
PKM<br>*(Lightning link)*	BSA/Azide free<br>PE	Abcam	ab206129<br>ab102918
SDHA<br>*(Lightning link)*	BSA/Azide free<br>AF647	Abcam	ab240098<br>ab269823
CPT1A<br>*(Lightning link)*	BSA/Azide free<br>PE/Cy5	Abcam	ab235841<br>ab102893
ACC1<br>*(Lightning link)*<br>*(Lightning link)*	BSA/Azide free<br>AF488<br>PE/Cy7	Abcam	ab272704<br>ab236553<br>ab102903
Cytc<br>*(Lightning link)*	BSA/Azide free<br>PE/Cy7	Abcam	ab237966<br>ab102903
ATP5a<br>*(Lightning link)*	BSA/Azide free<br>AF488	Abcam	ab231692<br>ab236553
G6PD<br>*(Lightning link)*	BSA/Azide free<br>APC/Cy7	Abcam	ab231828<br>ab102859
FDFT1	DL550	Novusbio<br>Abcam	NBP2-70715R
CS<br>*(Lightning link)*	BSA/Azide free<br>AF488	Abcam	ab233838<br>ab236553
ATGL<br>*(Lightning link)*	BSA/Azide free<br>AF647	Abcam	ab272317<br>ab269823
ACADM<br>*(Lightning link)*	BSA/Azide free<br>DL405	Abcam	ab239914<br>ab201798

Table S3: Primers used for quantitative PCR

Gene	Forward 5' – 3'	Reverse 3' – 5'
β2-microglobulin	GCCTGCCGTGTGAACCAT	TTACATGTCTCGATCCCACTTAACTAT
GAPDH	CAGCCTCAAGATCATCAGCA	TGTGGTCATGAGTCCTTCCAA
AMPKα1	TCGGCAAAGTGAAGGTTGGCA	TCCTACCACACTCAAGGCTCCGAA
PPARγ	CTCATATCCGAGGGCCAA	TGCCAAGTCGCTGTCATC
PGC1α	ACTGCAGGCCTAACTCCACCCA	ACTCGGATTGCTCCGGCCCT

Supplemental auxiliary files

Data File S1: Metabolomics of RA-DCs
